# Spatiotemporal Dynamics of Cytosolic NADPH in Living *Arabidopsis thaliana*

**DOI:** 10.1111/pce.70806

**Published:** 2026-08-11

**Authors:** Danying Lu, Jinhong Liu, Shey‐Li Lim, Jia Yi Zhong, Boon Leong Lim

**Affiliations:** ^1^ School of Biological Sciences University of Hong Kong Hong Kong China; ^2^ HKU Shenzhen Institute of Research and Innovation Shenzhen China; ^3^ State Key Laboratory of Agrobiotechnology The Chinese University of Hong Kong Hong Kong China

**Keywords:** Arabidopsis, cytosol, NADPH, NADP‐ICDH, NADP‐ME, np‐GADPH, oxidative pentose phosphate pathway

## Abstract

In plants, an adequate supply of Nicotinamide adenine dinucleotide phosphate (NADPH) in the cytosol is crucial for maintaining a variety of biosynthetic and antioxidant reactions. Cytosolic NADPH is mainly generated via four enzymatic pathways: NADP‐dependent malic dehydrogenase (NADP‐ME), cytosolic oxidative pentose phosphate pathway (cOPPP), cytosolic NADP‐dependent isocitrate dehydrogenase (cyNADP‐ICDH), and non‐phosphorylating glyceraldehyde‐3‐phosphate dehydrogenase (np‐GAPDH). Here, we employed the cytosolic NADPH sensor mCherry‐iNAP1 along with a GUS reporter system and enzyme assays to clarify the contributions of the four NADP(H)‐reducing pathways in Arabidopsis seedlings and mature leaves. Our results show that, in 5‐day‐old seedlings, cyNADP‐ICDH and np‐GAPDH supply cytosolic NADPH to both roots and shoots, cOPPP could contribute more cytosolic NADPH to roots than to shoots, and NADP‐ME2 primarily functions in roots. In leaves of 21‐day‐old plants, cyNADP‐ICDH and np‐GAPDH supply cytosolic NADPH throughout the day, while cOPPP could be more active at night, and NADP‐ME2 utilizes daytime‐accumulated malate to produce substantial cytosolic NADPH at night. Spatiotemporal visualization of cytosolic NADPH through *in vivo* imaging provides crucial information for future redox engineering of stress‐resistant crops.

## Summary statement

In 5‐day‐old seedlings of *Arabidopsis thaliana*, cytosolic NADP‐dependent isocitrate dehydrogenase (cyNADP‐ICDH) and non‐phosphorylating glyceraldehyde‐3‐phosphate dehydrogenase (np‐GAPDH) supply cytosolic NADPH to both roots and shoots, cytosolic oxidative pentose phosphate pathway (cOPPP) could contribute more cytosolic NADPH to roots than to shoots, and NADP‐dependent malate dehydrogenase 2 (NADP‐ME2) primarily functions in roots. In leaves of 21‐day‐old Arabidopsis plants, cyNADP‐ICDH and np‐GAPDH supply cytosolic NADPH throughout the day, while cOPPP is more active at night, and NADP‐ME2 utilizes daytime‐accumulated malate to produce substantial cytosolic NADPH at night.

## 1 Introduction

Nicotinamide adenine dinucleotide phosphate (NADPH) is a key electron carrier involved in redox homeostasis regulation and multiple anabolic pathways. In illuminated chloroplasts, NADPH is primarily regenerated from reducing NADP^+^ via photosynthesis, while in darkness and in non‐green plastids, the plastidic oxidative pentose phosphate pathway (OPPP) is considered as the major source of stromal NADPH (Emes and Neuhaus 1997; Wakao et al. 2008). Under light conditions, NADPH level in the chloroplast stroma is significantly higher than those in the other cellular compartments, and cytosolic NADPH levels are substantially lower than those in the chloroplast stroma and peroxisomes (Lim et al. 2020). Stromal NADPH generated from photosynthesis cannot diffuse directly across organelle membranes into the cytosol, and the existence of a chloroplast NADPH transporter has yet to be validated (Heineke et al. 1991; Lu et al. 2025). Instead, the excess reducing equivalents carried by stromal NADPH can be channeled to the cytosol in the form of malate, which is subsequently oxidized to generate cytosolic NADPH by NADP^+^‐reducing enzymes (Lim et al. 2020).

In Arabidopsis, cytosolic NADPH can serve as a substrate for key enzymes to participate in reactive oxygen species (ROS) metabolism, stress response and regulate cellular redox balance, including respiratory burst oxidase homolog (RBOH, AtRboHA‐J) (Chapman et al. 2019), cytosolic glutathione reductase AtGR1 in cytosolic ascorbate‐glutathione (AsA‐GSH) cycle (Noctor et al. 2012), cytosolic NADPH‐dependent thioredoxin reductase AtNTRA (Cha et al. 2015), and alternative NADPH dehydrogenases AtNDB1 located on the outer side of the mitochondrial inner membrane (Rasmusson et al. 2020). In photorespiration, cytosolic hydroxypyruvate reductase AtHPR2 constitutes a cytosolic bypass, in addition to the peroxisomal pathway catalyzed by NADH‐dependent AtHPR1, that catalyzes the conversion of hydroxypyruvate to glycerate by consuming NADPH (Cousins et al. 2008; Timm et al. 2008). Cytosolic NADPH can also be consumed by β‐Keto‐acyl‐CoA reductase (KCR) and enoyl‐CoA reductase (ECR) subunits in the fatty acid elongation (FAE) complex anchored on the cytosolic surface of the endoplasmic reticulum (ER), thereby participating in the synthesis of very‐long‐chain fatty acids (VLCFAs) (Batsale et al. 2021). In plants, VLCFAs serve as crucial precursors for biosynthetic pathways including cell membranes, cuticular waxes, and root suberin (Batsale et al. 2021). Additionally, the ER membrane‐anchored cytochrome P450 reductase AtATR1 transfers electrons from cytosolic NADPH to downstream cytochrome P450 enzymes, such as cinnamic acid 4‐hydroxylase (C4H) anchored to the ER membrane, to drive hydroxylation reactions that are essential for precursors generation of lignin and flavonoids (Achnine et al. 2004; Sundin et al. 2014). All these studies highlight the crucial role of NADPH as an electron donor for numerous essential cytosolic pathways in plant cells. Despite this, the study of cytosolic NADPH supply in Arabidopsis seedlings and mature leaves has received little attention.

In the cytosol, NADPH can be generated via four major NADP^+^‐reducing pathways, including NADP‐dependent malic enzymes (NADP‐ME), the cytosolic OPPP (cOPPP), NADP‐dependent isocitrate dehydrogenases (NADP‐ICDH), and non‐phosphorylated glyceraldehyde‐3‐phosphate dehydrogenase (np‐GAPDH). NADP‐MEs catalyze the oxidative decarboxylation of malate and generate pyruvate, CO_2_, and NADPH. In Arabidopsis, there are four NADP‐MEs, cytosolic AtNADP‐ME1‐3, and plastidic AtNADP‐ME4 (Wheeler et al. 2005). In the cOPPP, glucose‐6‐phosphate dehydrogenase (G6PDH) and 6‐phosphogluconate dehydrogenase (6PGDH) convert one molecule of glucose‐6‐phosphate (G6P) to ribulose‐5‐phosphate (Ru5P) and generate two molecules of NADPH (Wakao et al. 2008). In Arabidopsis, there are six G6PDH isoforms, among which AtG6PD1‐4 are located in plastids, AtG6PD6 is localized in the cytosol, and the five splice forms of AtG6PD5, AtG6PD5.1‐5.5, have different localizations. AtG6PD5.1‐5.3 are localized in the cytosol, AtG6PD5.4 and AtG6PD5.5 are anchored to the cytosolic surface of the ER membrane with the protein activity of AtG6PD5.4 in protoplast extract was significantly higher than that of AtG6PD5.5 (Linnenbrügger et al. 2022; Linnenbrügger et al. 2025; Wakao and Benning 2005). Despite their predominant plastidic localization, a fraction of AtG6PD1 and AtG6PD4 can be found in the cytosol, where AtG6PD4 interacts with the AtG6PD1 precursor and facilitates its peroxisomal import to complete the peroxisomal OPPP (Corpas et al. 1998; Meyer et al. 2011). Three Arabidopsis 6PGDH isoforms are found in the Arabidopsis genome, among which AtPGD1 and AtPGD3 are dual localized to the cytosol and in plastids, and AtPGD2 is dual targeted to the cytosol and peroxisomes (Hölscher et al. 2016; Lutterbey and von Schaewen 2016). NADP‐ICDHs dehydrogenate isocitrate to 2‐oxygluconate (2‐OG) and generate NADPH (Mhamdi et al. 2010). Arabidopsis has three NADP‐ICDH isoforms: the mitochondrial/chloroplastic NADP‐ICDH encoded by the same gene, cytosolic NADP‐ICDH, and peroxisomal NADP‐ICDH (Corpas et al. 1999; Leterrier et al. 2016). np‐GAPDH, a member of the aldehyde dehydrogenase superfamily, mainly found in autotrophic eukaryotes (e.g., vascular plants), catalyzes the oxidation of glyceraldehyde‐3‐phosphate (GAP) to 3‐phosphoglycerate in the cytosol and generates one molecule of NADPH (Bustos and Iglesias 2003; Wieloch 2021).

Of the four major cytosolic NADP^+^‐reducing pathways, it remains unclear which enzyme(s)/pathway(s) are major suppliers of cytosolic NADPH in roots and shoots. Furthermore, it remains elusive whether cytosolic NADPH supply pathways differ under light and dark conditions in mature leaves. Previous in vitro methods for measuring energy cofactors in plant cells mostly require prior extraction of these cofactors, which would easily distort the actual amount of pyridine nucleotides. By analyzing the tissue‐specific expression patterns of cytosolic NADP^+^‐reducing enzyme‐encoding genes via a GUS reporter system, comparing the enzyme activities of NADP‐ME, G6PDH, NADP‐ICDH, and np‐GAPDH in leaf cytosolic fractions from WT and mutants under both light and dark conditions, and introducing a novel fluorescent sensor mCherry‐iNAP1 for *in planta* NADPH level measurement into wild‐type (WT, Col‐0) Arabidopsis and several mutants (Lim et al. 2024), we clarified the sources of cytosolic NADPH in young seedlings and mature leaves.

## 2 Materials and Methods

### 2.1 Plant Growth Conditions

Arabidopsis seeds were sterilized in a 20% (v/v) bleach (Clorox^TM^, Malaysia) solution for 15 min, washed with sterile water, and then germinated onto Murashige and Skoog (MS) solid medium (pH 5.7–5.8, supplemented with 2% (w/v) sucrose, 0.25% (w/v) Phytagel, 0.1% (w/v) 2‐(*N*‐morpholino) ethanesulfonic acid, and corresponding antibiotics) (Murashige and Skoog 1962). The sterilized Arabidopsis seeds in MS plates were stratified at 4°C in darkness for 3 days before transferred to the tissue culture room. Ten days after seed germination on MS medium, seedlings were transplanted into soil and cultured in growth chambers under conditions of 16 h light/8 h dark at 22°C/18°C (day/night).

### 2.2 Promoter‐GUS Fusion Construction and Generation of Transgenic Plant Lines

To generate promoter‐GUS fusion constructs, the original GUS vector pBI121*::35S*‐GUS (https://www.snapgene.com/plasmids/plant_vectors/pBI121?set=plant_vectors&plasmid=pBI121) was digested with HindIII and BamHI restriction enzymes (New England BioLabs, England). Based on the TAIR website (https://www.arabidopsis.org/), the promoter sequences of *AtNADP‐ME1*, *AtNADP‐ME2*, *AtNADP‐ME3*, *AtG6PD5*, *AtPGD1*, *AtPGD2*, *AtPGD3*, and *Atnp‐GAPDH* were amplified from Arabidopsis genomic DNA using Phanta DNA polymerase (Vazyme Biotech, China) following the standard PCR protocol. The amplified PCR products were then confirmed by sequencing before cloned into the digested pBI121*::35S*‐GUS using Exnase II (Vazyme Biotech, China). Primer sequences used for promoter cloning are listed in Table S5. The pEG*::AtG6PD6*‐GUS (Figure S17) and pEG*::AtcyNADP‐ICDH*‐GUS (Figure S18) were acquired from EDGENE BIO company, Wuhan, China.

The promoter‐GUS fusion constructs pBI121*::AtNADP‐ME1*‐GUS, pBI121*::AtNADP‐ME2*‐GUS, pBI121*::AtNADP‐ME3*‐GUS, pBI121*::AtPGD1*‐GUS, pBI121*::AtPGD2*‐GUS, pBI121*::AtPGD3*‐GUS, pBI121*::AtG6PD5*‐GUS, pBI121*::Atnp‐GAPDH*‐GUS, pEG*::AtG6PD6*‐GUS and pEG*::AtcyNADP‐ICDH*‐GUS were transferred into *Agrobacterium tumefaciens* strain GV3101 and introduced into Col‐0 Arabidopsis via the floral dip method (Clough and Bent 1998). The positive transformants were screened on selective medium supplemented with kanamycin (50 mg/L) or basta (25 mg/L) antibiotics for *pBI121‐GUS* transgenic plants or *pEGpro‐GUS* transgenic plants, respectively.

### 2.3 Gus Histochemical Staining

The 1‐day‐old, 2‐day‐old, 5‐day‐old, and 10‐day‐old seedlings germinated on MS medium, and leaves from 21‐day‐old plants were collected for GUS histochemical staining experiments. The plant samples were first immersed in 90% (v/v) acetone (4°C) for 20 min and then rinsed three times using washing buffer (0.1% (v/v) Triton X‐100, 20% (v/v) methanol, 8 mM EDTA‐Na_2_, 0.4 mM K_3_[Fe(CN)_6_], 0.4 mM K_4_[Fe(CN)_6_]·3H_2_O, 80 mM Na_2_HPO_4_·12H_2_O (pH 7.0), and 80 mM NaH_2_PO_4_·H_2_O (pH7). Subsequently, plant samples were stained with X‐Gluc solution (0.8 mg/ml X‐Gluc, 20% (v/v) methanol, 0.1% (v/v) Triton X‐100, 8 mM EDTA‐Na_2_, 0.4 mM K_3_[Fe(CN)_6_], 0.4 mM K_4_[Fe(CN)_6_]·3H_2_O, 80 mM Na_2_HPO_4_·12H_2_O (pH 7.0), and 80 mM NaH_2_PO_4_·H_2_O (pH 7.0) on ice, vacuum‐infiltrated for 20 min, and then incubated in the dark at 37°C for 6 h. Finally, these samples were destained with 70% (v/v) ethanol, and images were acquired using an Olympus SZX 16 stereo fluorescent microscope.

### 2.4 T‐DNA Mutants Verification

The sources of T‐DNA insertion mutant seeds used in this study were as follows: *nadp‐me1* (SALK_036898), *nadp‐me2* (SALK_020607), and *nadp‐me3* (SALK_139336) were kindly provided by Professor Bentsink at Wageningen University (Yazdanpanah et al. 2019); *pgd1* (GABI_762C06), *cynadp‐icdh* (SALK_056247), and *np‐gapdh* (SAIL_1152_B02) were purchased from the ABRC center (https://abrc.osu.edu/). The T‐DNA insertion sites and knock out of target genes in *nadp‐me1*, *nadp‐me2*, *nadp‐me3* (Wheeler et al. 2005), *pgd1* (Andriotis and Smith 2019), and *cynadp‐icdh* (Mhamdi et al. 2010) have been reported and validated previously. The T‐DNA insertion site of *np‐gapdh* was confirmed by sequencing PCR amplification products (service performed by BGI Genomics, China). The knock out of *Atnp‐GAPDH* in *np‐gapdh* mutant was validated by semi‐quantitative PCR. Homozygous mutants for each line were determined by genotyping, and the genotyping primers were designed using the SIGnAL online tool (http://signal.salk.edu/tdnaprimers.2.html). Primers used for semi‐PCR and genotyping were all listed in Table S5.

### 2.5 Mutant Generation By a CRISPR‐CAS9 System

The *g6pd5.4*, *g6pd6*, and *pgd3* mutants were all generated using a CRISPR/Cas9 system. Single‐guide RNAs (sgRNAs) for each gene were designed using the online tool CCTop (http://crispr.cos.uni-heidelberg.de/) and listed in Table S5. The sgRNA was cloned individually into the pKI1.1R vector (https://www.addgene.org/85808/), which contains the Arabidopsis RIBOSOMAL PROTEIN S5A (AtRPS5A) promoter driving Cas9 and the OLE1‐TagRFP reporter gene (Tsutsui and Higashiyama 2017). Then, the pKI1.1R harboring the sgRNA was transformed into Col‐0 plants using the floral dip method. The T1 generation seeds were selected on medium supplemented with 50 mg/L hygromycin. To determine *g6pd5.4*, *g6pd6*, and *pgd3* mutants, the targeted sequences were cloned using Phanta Max Super‐Fidelity DNA Polymerase (Vazyme, China) and sequenced by BGI Genomics. The primers used for PCR and sequencing were listed in Table S5. Subsequently, the Cas9‐free seeds without red fluorescence in the T2 generation were screened using a Nikon SMZ18 fluorescence stereomicroscope (Nikon, Japan). The Cas9‐free *g6pd5.4*, *g6pd6*, and *pgd3* mutants were used for the experiments, and the *g6pd5.4/6* double mutant was generated by crossing of *g6pd5.4* and *g6pd6* plants.

### 2.6 Generation of Mutants Expressing mCherry‐iNAP1

The seeds of WT *mCherry‐iNAP1* and pH7WG2 vector carrying mCherry‐iNAP1 were generated previously (Lim et al. 2024). The fluorescence ratio of mCherry‐iNAP1 remains stable within the pH range of 7.0‐8.5 (Lim et al. 2024; Liu et al. 2022). The pH7WG2 vector carrying mCherry‐iNAP1 was transformed into *nadp‐me1*, *nadp‐me2*, *nadp‐me3*, *pgd1*, *pgd3*, *g6pd5.4*, *g6pd6*, *g6pd5.4/6*, *cynadp‐icdh*, and *np‐gapdh* plants in bloom using the floral dip method. Positive transformants expressing mCherry‐iNAP1 were identified using an Olympus SZX16 fluorescence stereomicroscope.

### 2.7 Plant Treatment

In Figure 1a, before image acquisition, 5‐day‐old WT *mCherry‐iNAP1* seedlings in the dark group were adapted in dark for 2 h, and plants in the light group were kept in light. In Figure 1e, confocal images of all the rosette leaves of 21‐day‐old WT *mCherryiNAP1* plants in light group were acquired at the end of the day, 16 h after the start of illumination, and in the middle of the night in dark group, 3–4 h after the onset of darkness. In Figure 2, before image acquisition, 5‐day‐old WT and mutants expressing mCherry‐iNAP1 were under light conditions. In Figure 3, confocal images of the third or fourth leaves of 21‐day‐old WT and mutants expressing mCherry‐iNAP1 were captured at the end of the day. In Figure 4, confocal images of the fully expanded leaves of 21‐day‐old WT and mutants expressing mCherry‐iNAP1 were collected at the end of the day in light group and in the middle of the night in dark group.

**FIGURE 1:**
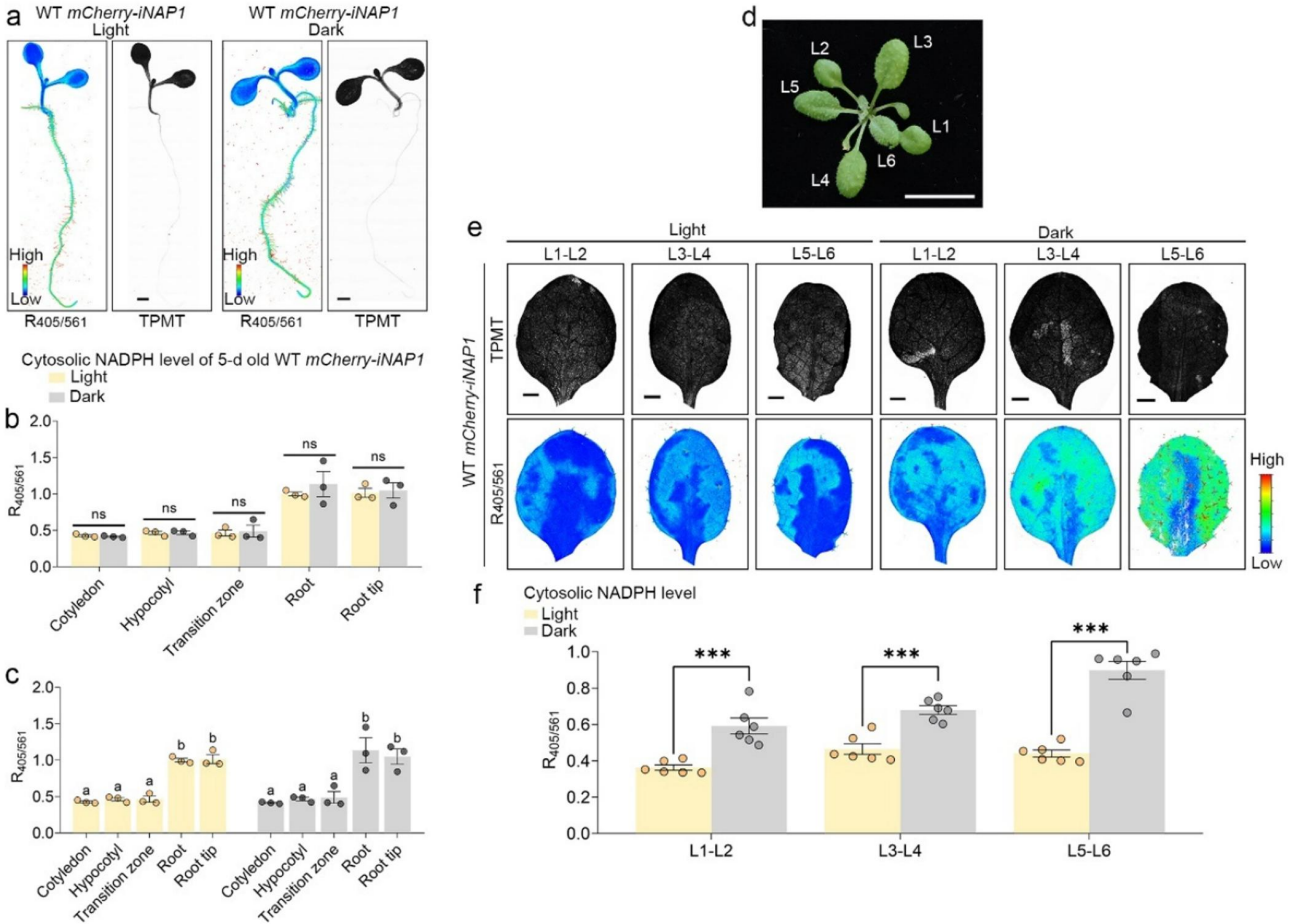
Cytosolic NADPH levels in WT *mCherry‐iNAP1* seedlings under light and dark conditions. (a) Ratio images and bright‐field images of 5‐day‐old WT *mCherry‐iNAP1* seedlings subjected to light/dark conditions. Scale bar = 1000 μm. Comparation of R_405/561_ ratios in (b) same tissue and (c) different tissues of 5‐day‐old WT *mCherry‐iNAP1* seedlings under light/dark conditions. For each condition, *n* = 3 seedlings, error bars ± SEM. Figure panels b and c are derived from the same dataset. (d) Leaves of a representative 21‐day‐old WT Arabidopsis plant were numbered from older expanded leaves (leaves 1–2, L1–L2), fully expanded leaves (leaves 3–4, L3–L4), to newly grown leaves (leaves 5–6, L5–L6). (e) Ratio images and bright‐field images of leaves of 21‐day‐old WT *mCherry‐iNAP1* seedlings under light and dark conditions. Scale bar = 1000 μm. Comparation of R_405/561_ ratios in (f) same‐aged leaves under light and dark conditions. In each column, *n* = 6 leaves from three different seedlings, error bars ± SEM. Fluorescence from sequential excitation wavelength at 405 and 561 nm, and the emission was collected at 520 ± 16 nm or 609 ± 25 nm, respectively. Significant differences of R_405/561_ ratios in panel b and f were determined by an independent samples *t*‐test (three asterisks for *p* < 0.001, two asterisks for *p* < 0.01, and one asterisks for *p* < 0.05), and in (c) were determined by One‐way ANOVA with Tukey's HSD (indicated with different letters, *p* < 0.05). ns, not significant. All ratio images are presented in pseudocolor where low R_405/561_ ratios (blue) correspond to low NADPH levels and high R_405/561_ ratios (red) correspond to high NADPH levels. TPMT, transmitted light detector; WT, wild‐type.

**FIGURE 2:**
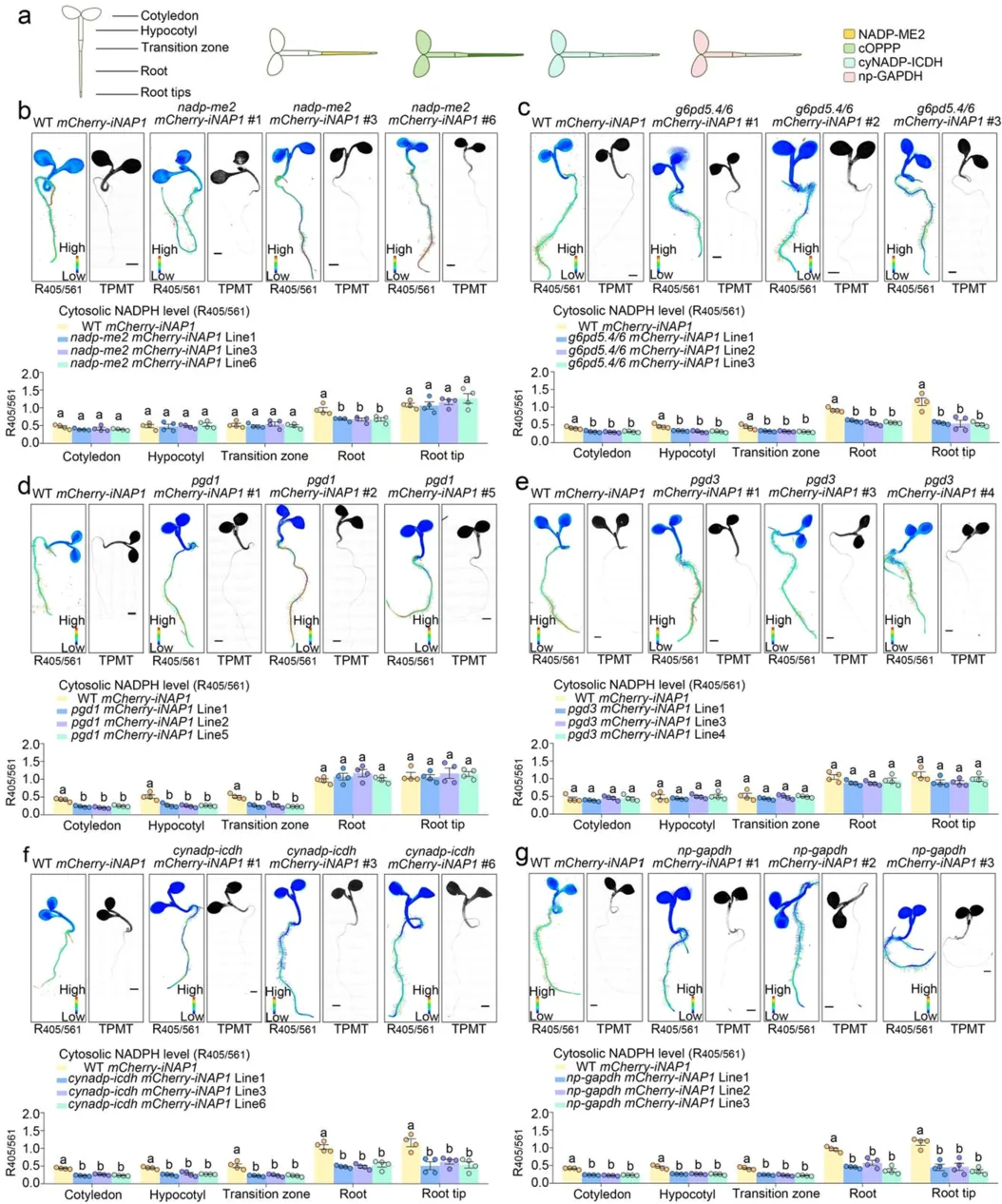
R_405/561_ ratios in 5‐day‐old WT, *nadp‐me2*, *g6pd5.4/6*, *pgd1*, *pgd3*, *cynadp‐icdh* and *np‐gapdh* seedlings constitutively expressing *mCherry‐iNAP1* under illumination. (a) Schematic diagram of sources of cytosolic NADPH in 5‐day‐old Arabidopsis seedlings. The colored tissues indicate the functional areas of specific cytosolic NADPH sources. NADP‐ME2, NADP‐dependent malic enzyme 2; cOPPP, cytosolic oxidative pentose phosphate pathway; cyNADP‐ICDH, cytosolic NADP‐dependent isocitrate dehydrogenase; np‐GAPDH, non‐phosphorylating glyceraldehyde‐3‐phosphate dehydrogenase. Comparison of ratio images and bright‐field images, and R_405/561_ ratios of 5‐day‐old (b) *nadp‐me2*, (c) *g6pd5.4/6*, (d) *pgd1*, (e) *pgd3*, (f) *cynadp‐icdh* or (g) *np‐gapdh* seedlings expressing *mCherry‐iNAP1* with WT *mCherry‐iNAP1* plants. Three independent lines expressing *mCherry‐iNAP1* were characterized for each mutant genotype. In each line, R_405/561_ ratios of 5 different tissues from 4 seedlings (*n* = 4) were subjected to data analysis, errors bar ± SEM, scale bar = 1000 µm. Fluorescence from sequential excitation wavelength at 405 and 561 nm, and the emission was collected at 520 ± 16 nm or 609 ± 25 nm, respectively. Ratio images are presented in pseudocolor, with red and blue representing high and low R_405/561_ ratios (indicative of high and low NADPH levels), respectively. The significant differences in cytosolic NADPH levels were determined by One‐way ANOVA with Tukey's HSD (*p* < 0.05) and indicated with different letters. TPMT, transmitted light detector; WT, wild‐type.

**FIGURE 3:**
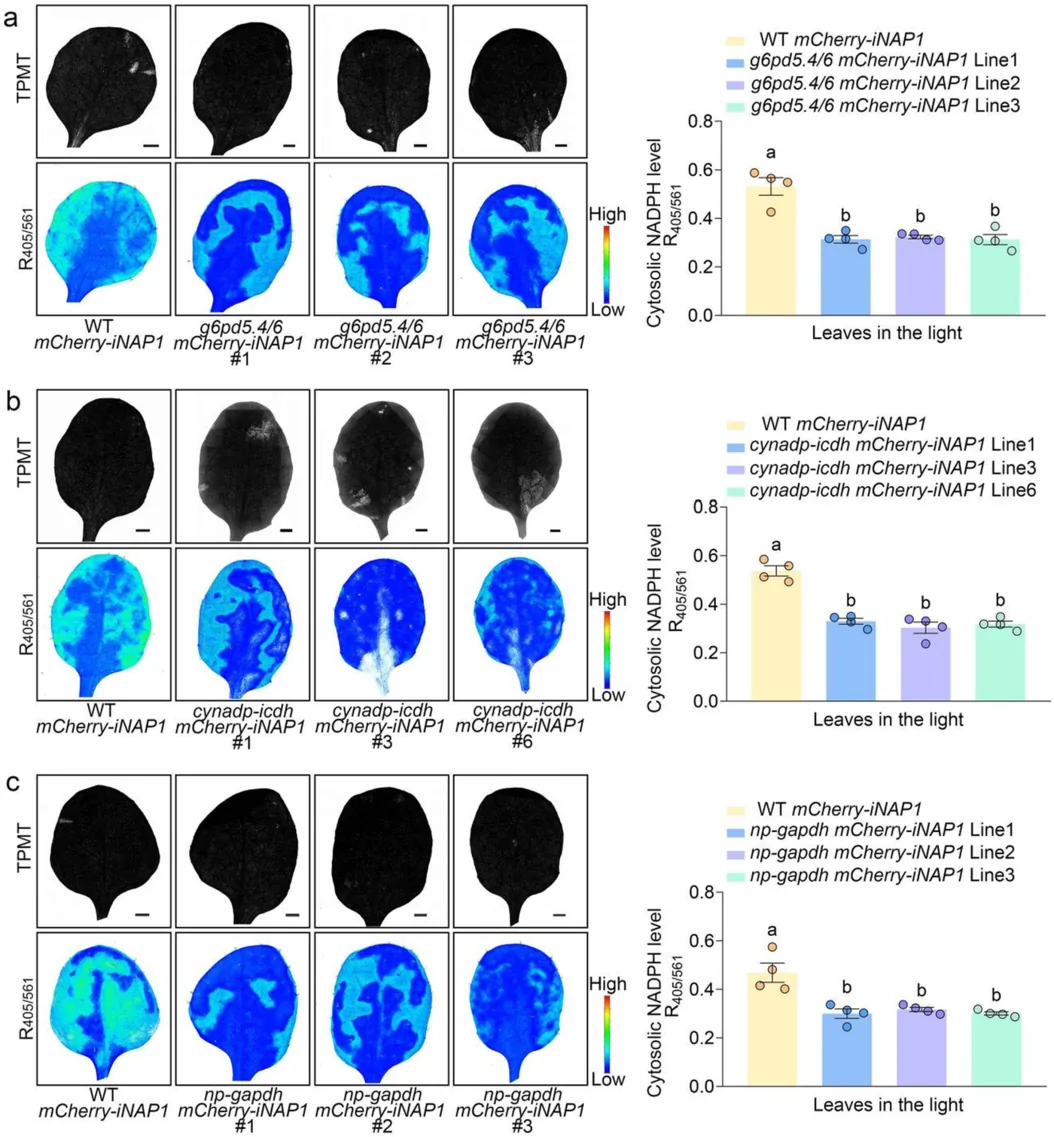
R_405/561_ ratios in fully expanded leaves of 21‐day‐old WT, *g6pd5.4/6*, *cynadp‐icdh*, *np‐gapdh* seedlings constitutively expressing *mCherry‐iNAP1* in the light. Comparison of ratio images and bright‐field images, and R_405/561_ ratios of fully expanded leaves in 21‐day‐old (a) *g6pd5.4/6*, (b) *cynadp‐icdh*, or (c) *np‐gapdh* seedlings expressing *mCherry‐iNAP1* with WT *mCherry‐iNAP1* plants in the light. Three independent lines expressing mCherry‐iNAP1 were characterized for each mutant genotype. For each column, *n* = 4 fully expanded leaves (L3 or L4) from four different seedings, errors bar ± SEM, scale bar = 1000 µm. Fluorescence from sequential excitation wavelength at 405 and 561 nm, and the emission was collected at 520 ± 16 nm or 609 ± 25 nm, respectively. Ratio images are presented in pseudocolor, with red and blue representing high and low R_405/561_ ratios (indicative of high and low NADPH levels), respectively. The significant differences in cytosolic NADPH levels were determined by One‐way ANOVA with Tukey's HSD (*p* < 0.05) and indicated with different letters. TPMT, transmitted light detector; WT, wild‐type.

**FIGURE 4:**
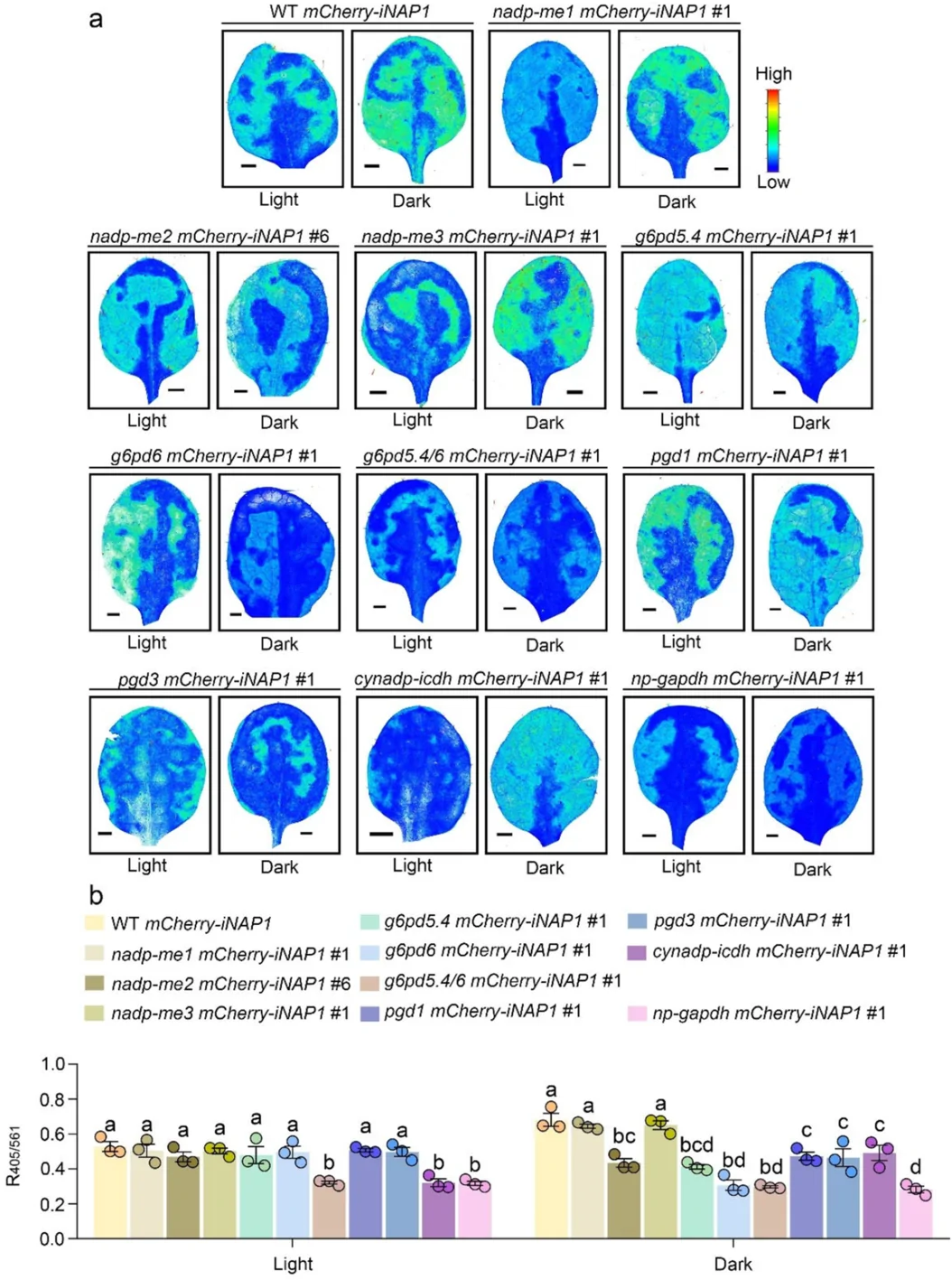
R_405/561_ ratios in fully expanded leaves of 21‐day‐old WT and mutants constitutively expressing *mCherry‐iNAP1* under light and dark conditions. (a) Ratio images of fully expanded leaves in 21‐day‐old WT and mutants seedlings expressing mCherry‐iNAP1 under light and dark conditions. Scale bar = 1000 μm. Ratio images are presented in pseudocolor, with red and blue representing high and low R_405/561_ ratios (indicative of high and low NADPH levels), respectively. (b) Comparation of R_405/561_ ratios in WT and mutants expressing mCherry‐iNAP1 under light and dark conditions. As the R_405/561_ ratios of the three mCherry‐iNAP1 transgenic lines of each mutant were relatively consistent, one line expressing mCherry‐iNAP1 for each mutant was selected to compare the R_405/561_ ratios with WT mCherry‐iNAP1 leaves under light or dark conditions. In each column, *n* = 3 fully expanded leaves (L3 or L4) from three different seedlings, error bars ± SEM. Significant differences of R_405/561_ ratios were determined by One‐way ANOVA with Tukey's HSD (indicated with different letters, *p* < 0.05).

### 2.8 Confocal Microscopy and Image Analysis

Confocal images were captured using a Zeiss LSM 980 microscope (Carl Zeiss, Germany) with ×5/0.16 Ph1 objective in multitrack mode. Seedlings expressing mCherry‐iNAP1 were sequentially excited at 405 and 561 nm, with emission detected at 520 ± 16 and 609 ± 25 nm, respectively. Autofluorescence was excited at 405 nm and emission detected at 450 ± 17 nm. Chlorophyll fluorescence was excited at 488 nm with emission detected at 670 ± 30 nm (Lim et al. 2024). The mCherry‐iNAP1 ratio, representing the ratio of emission excited at 405 and 561 nm, is denoted as R_405/561_. Confocal images were processed using a custom MATLAB‐based suite (Fricker 2016). The ratiometric images underwent a pixel‐by‐pixel analysis, and all ratio images were shown in pseudocolors (Lim et al. 2024).

### 2.9 Enzyme Assays

Rosette leaves from 21‐day‐old WT, *nadp‐me2*, *g6pd5.4/6*, *cynadp‐icdh*, and *np‐gapdh* seedlings were used for the extraction of cytosolic fractions (Keech et al. 2005). Three biological replicates were collected from each line under either dark or light conditions. For each biological replicate, 1 g of rosette leaves was harvested and homogenized by Polytron blender in 15 mL of cold grinding medium. The grinding medium consisted of 0.3 M sorbitol, 50 mM Tris‐HCl (Tris(hydroxymethyl)aminomethane hydrochloride, pH 7.5), 10 mM KH_2_PO_4_, and 2 mM ethylenediaminetetraacetic acid (EDTA), adjusted to pH 7.5 with 1 M KOH. To prevent protein degradation, 1 mM phenylmethanesulfonyl fluoride (PMSF) was added to the grinding medium prior to use. The homogenized leaves were filtered through cheesecloth and centrifuged at 15,000 *g* (JA‐20 rotor) at 4°C for 15 min. The crude cytosolic fraction in the supernatant was used for subsequent enzyme activity assays.

All enzyme activities were determined spectrophotometrically at 340 nm and 24°C. Protein contents were determined using the Bradford method (Bradford 1976). One enzyme activity unit (U) is defined as the amount of enzyme required to catalyze the formation of 1 μmol NADPH per minute under specific assay conditions. Six technical replicates were performed per experiment per biological replicate, and the final volume of each reaction was 0.2 mL. NADP‐ME activity was determined using a standard reaction mixture consisting of 50 mM Tris‐HCl (pH 7.5), 10 mM MgCl_2_, 0.4 mM NADP, and 10 mM l‐malate (Wheeler et al. 2005). The reaction was initiated by the addition of l‐malate. The method for determining G6PDH activity was adapted from previous researches (Graeve et al. 1994; Hauschild and von Schaewen 2003). G6PDH activity was measured in a buffer consisting of 0.1 M Tris‐HCl (pH 8.0), 0.4 mM NADP, 62.5 mM dithiothreitol (DTT), and 2 mM G6P. The reaction was initiated by the addition of G6P. NADP‐ICDH activity was measured using a reaction buffer consisting of 100 mM potassium phosphate (pH 7.5), 5 mM MgC1_2_, 2.5 mM DL‐isocitrate, and 0.4 mM NADP (Galvez et al. 1994). The reaction was initiated by the addition of isocitrate. The standard reaction mixture for the np‐GAPDH activity assay contained 50 mM Tricine‐NaOH (pH 8.5), 0.4 mM NADP, 1.2 mM fructose‐1,6‐bisphosphate, and 1 U/mL of aldolase (Rius et al. 2006).

### 2.10 Data Analysis

The collected data were plotted using GraphPad Prism (version 8) and were shown as means ± standard error (SEM). Statistical significance was assessed between the compared groups using an independent samples t‐test or analysis of variance (one‐way ANOVA, Tukey's HSD) at *p* < 0.001, *p* < 0.01, and *p* < 0.05 by SPSS (version 27). The schematic figure was created with BioGDP (www.biogdp.com) (Jiang et al. 2025).

### 2.11 Accession Numbers

Sequence data used in this study were obtained in the Arabidopsis Information Resource (https://www.arabidopsis.org) under the following accession numbers: *AtNADP‐ME1* (AT2G19900), *AtNADP‐ME2* (AT5G11670), *AtNADP‐ME3* (AT5G25880), *AtG6PD5* (AT3G27300), *AtG6PD6* (AT5G40760), *AtPGD1* (AT1G64190), *AtPGD2* (AT3G02360), *AtPGD3* (AT5G41670), *AtcyNADP‐ICDH* (AT1G65930), and *Atnp‐GAPDH* (AT2G24270).

## 3 Results

### 3.1 Cytosolic NAPDH Levels in Arabidopsis Seedlings and Leaves in Light and Dark Conditions

To determine the effect of light on NADPH levels, we first performed a whole scan of 5‐day‐old WT *mCherry‐iNAP1* seedlings. As indicated by the fluorescence ratio of mCherry‐iNAP1, there were no significant differences in cytosolic NADPH levels between light and dark conditions in the cotyledons, hypocotyls, transition zone, roots, and root tips (Figure 1a,b). Notably, cytosolic NADPH levels in roots and root tips were significantly higher than those in shoots (cotyledons, hypocotyls, and the transition zone) (Figure 1c), consistent with our previous findings (Lim et al. 2024). In soil‐grown 21‐day‐old plants, cytosolic NADPH levels in mature expanded leaves (L1–L2), fully expanded leaves (L3–L4), and newly emerged leaves (L5–L6) were significantly higher under dark conditions than under illumination (Figure 1d–f). This aligns with a previous finding showing that cytosolic NADPH levels in the cotyledon cells of 10‐day‐old WT plants significantly decreased upon transition from darkness to light (Lim et al. 2020).

### 3.2 Generation, Identification and Phenotyping of Mutants

To investigate the source(s) of cytosolic NADPH in Arabidopsis, we first obtained and characterized multiple homozygous mutants of genes encoding cytosolic NADP^+^‐reducing enzymes. Through genotyping, we identified T‐DNA homozygous lines deficient in cytosolic NADP‐MEs [*nadp‐me1* (SALK_036898), *nadp‐me2* (SALK_020607), *nadp‐me3* (SALK_139336)], cyNADP‐ICDH [*cynadp‐icdh* (SALK_056247)], PGD1 [*pgd1* (GABI_762C06)], and np‐GAPDH [*np‐gapdh* (SAIL_1152_B02)] (Figure S1). Through genotyping and RT‐PCR, the loss of *Atnp‐GAPDH* in the *np‐gapdh* (SAIL_1152_B02) homozygous mutant was confirmed (Figure S1l–m). Sequencing of the PCR products confirmed that the insertion site of SAIL_1152_B02 was located in the fourth exon (Figure S1k). A previous study found that AtG6PD5.4 can recruit 6‐phosphogluconolactonase 2 (AtPGL2) and AtPGD2 to anchor on the cytosolic side of the ER membrane to constitute OPPP metabolon at the ER (Linnenbrügger et al. 2022). As *pgd2* and *pgd1/3* homozygous mutants were lethal (Andriotis and Smith 2019; Hölscher et al. 2016), in addition to *pgd1* T‐DNA line, the *g6pd5.4*, *g6pd6*, and *pgd3* were generated using a CRISPR/Cas9 system to investigate the role of cOPPP. The *g6pd5.4/6* double mutant was produced through crossing *g6pd5.4* with *g6pd6* mutants. The nucleotide insertion or deletion in the *g6pd5.4*, *g6pd6*, *g6pd5.4/6*, and *pgd3* mutants were confirmed by sequencing their PCR products (Figure S1o,q,s,u), and the functional proteins encoded by the target genes in these mutants were aborted (Figure S12).

Among these mutants, only the rosette diameters of *g6pd5.4/6* (Figure S1t,w) and *np‐gapdh* (Figure S1n,w) mutants were significantly smaller than that of WT, and the rosette diameters of the other mutants were comparable to those of WT (Figure S1). Similarly, the reduced rosette diameters of *g6pd5.4/6* compared with WT resembled the phenotype previously reported in *g6pd5/6* T‐DNA insertion lines (Ruan et al. 2022), and the growth of another *np‐gapdh* mutant (SALK_021831) was also delayed (Rius et al. 2006).

### 3.3 AtNADP‐ME2 Mainly Supply Cytosolic NADPH in Roots of 5‐Day‐Old Seedlings

Among the three isoforms of cytosolic NADP‐MEs, AtNADP‐ME1 and AtNADP‐ME3 do not supply substantial cytosolic NADPH in 5‐day‐old seedlings (Figure S13a,b). Although the GUS staining assay revealed that *AtNADP‐ME1* was highly expressed in roots and cotyledons of 5‐day‐old Arabidopsis plants (Figure S2c), there were no significant differences in cytosolic NADPH levels in shoots and roots of 5‐day‐old seedlings between *nadp‐me1* and WT (Figure S13a). Similarly, cytosolic NADPH levels in shoots and roots of 5‐day‐old *nadp‐me3* plants were also comparable to that of the WT (Figure S13b), which could be attributed to the limited transcript expression of *AtNADP‐ME3* in trichomes and trichome basal cells of 5‐day‐old plants (Figure S4c). Notably, cytosolic NADPH levels in roots of 5‐day‐old *nadp‐me2* seedlings were significantly reduced compared to WT, while those in root tips and shoots showed no change relative to WT, indicating that AtNADP‐ME2 serves as a source of cytosolic NADPH in roots but not in root tips and shoots (Figure 2a–b). This finding was consistent with strong transcript expression of *AtNADP‐ME2* in the root but not in root tips of 5‐day‐old seedlings indicated by GUS staining results (Figure S3c).

### 3.4 cOPPP Can Supply Cytosolic NADPH to Both Roots and Shoots of 5‐Day‐Old Seedlings

Despite GUS staining data showing that *AtG6PD5* primarily functioned in the root and was slightly expressed in root tips (Figure S5c), the cytosolic NADPH levels in roots and root tips of 5‐day‐old *g6pd5.4* mutants were significantly reduced compared to WT, indicating that AtG6PD5.4 is essential for cytosolic NADPH supply in roots (Figure S13c). The cytosolic NADPH levels in cotyledons, hypocotyls, transition zones, roots, and root tips of 5‐day‐old *g6pd6* and *g6pd5.4/6* mutants were all significantly reduced compared to WT, indicating that AtG6PD6 supplies cytosolic NADPH to shoots and roots (Figure 2c and Figure S13d). GUS staining data showed that *AtG6PD6* was moderately expressed in cotyledons, strongly expressed in roots and the transition zone, and weakly expressed in hypocotyls (Figure S6c).

GUS staining data indicate that *AtPGD1* and *AtPGD3* were moderately and weakly expressed in shoots of 5‐day‐old plants, respectively, and were both strongly expressed in roots and root tips (Figure S7c and Figure S9c). In 5‐day‐old *pgd1* mutants, cytosolic NADPH levels were significantly reduced in cotyledons, hypocotyls, and transition zones, while no changes were observed in roots and root tips compared to WT (Figure 2d), indicating that AtPGD1 plays a crucial role in shoots. In contrast, the cytosolic NADPH levels in cotyledons, hypocotyls, transition zones, roots, and root tips of 5‐day‐old *pgd3* mutant were not changed compared to WT (Figure 2e). The unchanged cytosolic NADPH level in 5‐day‐old *pgd3* mutants and roots of 5‐day‐old *pgd1* mutants can be attributed to the cytosolic redundancy of AtPGD1‐3 (Andriotis and Smith 2019; Lutterbey and von Schaewen 2016), and the reduced cytosolic NADPH levels in shoots of 5‐day‐old *pgd1* mutants could be due to the stronger transcript expression of *AtPGD1* than *AtPGD3* in the shoots of 5‐day‐old seedlings (Figure S7c and Figure S9c). These data indicate that cOPPP is an essential source of cytosolic NADPH for shoots and roots of 5‐day‐old seedlings.

### 3.5 AtcyNADP‐ICDH and Atnp‐GAPDH Contribute to Cytosolic NADPH Supply in 5‐Day‐Old Seedlings

In 5‐day‐old *cynapd‐icdh* plants, cytosolic NADPH levels were greatly reduced in roots, root tips, transition zones, cotyledons, and hypocotyls compared to WT (Figure 2f), suggesting that AtcyNADP‐ICDH serves as a key NADPH provider in these tissues. GUS data supported the critical role of AtcyNADP‐ICDH in seedlings, which showed that *AtcyNADP‐ICDH* has moderate expression in both the shoots and roots of seedlings (Figure S10c). Although GUS data indicate that *Atnp‐GAPDH* was highly expressed in shoots of seedlings and weakly expressed in the roots (Figure S11c), cytosolic NADPH levels were significantly reduced in the cotyledons, hypocotyls, transition zones, roots, and root tips of 5‐day‐old *np‐gapdh* plants compared with WT (Figure 2g), indicating that Atnp‐GAPDH plays a crucial role in cytosolic NADPH supply to both the shoots and roots of 5‐day‐old seedlings.

### 3.6 Identification of Cytosolic NADPH Sources in Leaves Under Illumination

GUS staining data indicate that among *AtNADP‐ME1‐3*, only *AtNADP‐ME2* was strongly expressed in leaves of 21‐day‐old plants (Figures S2–S4). Under light conditions, the NADP‐ME activity in the cytosolic fractions of the *nadp‐me2* mutant leaves was significantly reduced by ~94% to 3.0 ± 0.8 mU/mg, compared with 48.7 ± 3.0 mU/mg in WT leaves (Figure S16a). While at the end of the day, cytosolic NADPH levels in fully expanded leaves of *nadp‐me1*, *nadp‐me2*, and *nadp‐me3* 21‐day‐old seedlings showed no significant change compared to WT (Figure S14), indicating that AtNADP‐ME1‐3 is not the primary source of cytosolic NADPH in leaves during the day.

Furthermore, GUS staining results revealed that among the five genes encoding NADP^+^‐reducing enzymes in cOPPP, *AtG6PD5* and *AtG6PD6* exhibited low expression levels in leaves (Figures S5–S6), *AtPGD1* and *AtPGD3* were mainly expressed in young leaves (Figures S7, S9), and *AtPGD2* was ubiquitous expressed in all leaves of 21‐day‐old seedlings (Figure S8). At the end of the day, compared with WT, cytosolic NADPH levels in fully expanded leaves of *g6pd5.4*, *g6pd6*, *pgd1*, and *pgd3* 21‐day‐old mutants exhibited no marked difference (Figure S15), while those in leaves of 21‐day‐old *g6pd5.4/6* plants were significantly impaired (Figure 3a). These phenomena might be due to the redundancy of G6PD isoenzymes (Wakao et al. 2008) or PGD isoenzymes (Andriotis and Smith 2019; Lutterbey and von Schaewen 2016) in the cytosol, and therefore the individual loss of AtG6PD5.4, AtG6PD6, AtPGD1, or AtPGD3 in leaves has limited effect on cytosolic NADPH supply (Figure S15). In the light, the G6PDH activity in the cytosolic fractions of *g6pd5.4/6* leaves was reduced by ~43% to 33.9 ± 0.7 mU/mg compared with WT leaves at 59.7 ± 1.7 mU/mg (Figure S16b). The residual G6PDH activity detected in the cytosolic fraction of *g6pd5.4/6* leaves may arise from the proteins encoded by *AtG6PD5.1‐5.3* and *AtG6PD5.5* transcripts. As AtG6PD5.4 and AtG6PD6 are both essential for recruiting downstream AtPGL2 and AtPGD2 (Linnenbrügger et al. 2022; Linnenbrügger et al. 2025), knockout of both genes significantly reduced daytime cytosolic NADPH supply in leaves (Figure 3a, Figure 4b).

*AtcyNADP‐ICDH* was detectable at moderate levels across all rosette leaves of 21‐day‐old plants (Figure S10). In the light, the NADP‐ICDH activity in the cytosolic fractions of *cynadp‐icdh* leaves was reduced by ~96% of WT leaves (82.4 ± 6.2 mU/mg) to 3.0 ± 1.4 mU/mg (Figure S16c). At the end of the day, cytosolic NADPH levels in fully expanded leaves of 21‐day‐old *cynadp‐icdh* mutants exhibited a remarkable decrease in cytosolic NADPH levels compared to the WT (Figure 3b), indicating that AtcyNADP‐ICDH is an important diurnal source of cytosolic NADPH in leaves. GUS staining results revealed that *Atnp‐GAPDH* was highly expressed in all the rosette leaves of 21‐day‐old plants (Figure S11). The np‐GAPDH activity in the cytosolic fractions of *np‐gapdh* leaves was reduced by ~98% to 1.1 ± 0.3 mU/mg in the light compared with WT leaves (51.9 ± 5.8 mU/mg) (Figure S16d). In the day, cytosolic NADPH levels in fully expanded leaves of 21‐day‐old *np‐gapdh* mutants were significantly impaired compared with WT, indicating that Atnp‐GAPDH can supply substantial amounts of cytosolic NADPH to leaves under illumination (Figure 3c).

### 3.7 Cytosolic NADPH Generation Enhanced in Leaves in the Dark

In the middle of the night, cytosolic NADPH levels in fully expanded leaves of *nadp‐me1* and *nadp‐me3* 21‐day‐old mutants were comparable to those in WT (Figure 4), while those in fully expanded leaves of *nadp‐me2* 21‐day‐old mutants were markedly depleted compared with WT (Figure 4b), indicating that AtNADP‐ME2 is an important source of cytosolic NADPH in leaves during the night. Notably, there was no significant difference in cytosolic NADP‐ME activity between day and night in both WT and *nadp‐me2* leaves (Figure S16a), and cytosolic NADP‐ME activity of *nadp‐me2* leaves (1.9 ± 0.1 mU/mg) were reduced by ~96% of that of WT leaves (55.1 ± 1.6 mU/mg) in the night (Figure S16a), indicating that NADP‐ME capacity is not the primary factor causing the difference in NADPH supply from AtNADP‐ME2 between day and night. Similarly, under both light and dark conditions, there was no difference in G6PDH activity between day and night in the cytosolic fractions of WT and *g6pd5.4/6* leaves (Figure S16b). G6PDH activity of the cytosolic fractions of *g6pd5.4/6* leaves (31.8 ± 2.3 mU/mg) were reduced by ~50% of that of the WT leaves (63.6 ± 0.3 mU/mg) in the night (Figure S16b). In contrast to the daytime, at night, the cytosolic NADPH levels in fully expanded leaves of 21‐day‐old *g6pd5.4*, *g6pd6*, *g6pd5.4/6*, *pgd1*, and *pgd3* plants were all significantly reduced compared with the WT (Figure 4), showing that cOPPP could supply more cytosolic NADPH at night than during the day, and G6PDH capacities are not the main factor causing this difference. In the dark, cytosolic NADP‐ICDH activity and np‐GAPDH activity in the leaves of *cynadp‐icdh* and *np‐gapdh* leaves were both significantly reduced by over 90% than that of WT, and both NADP‐ICDH and np‐GAPDH activities did not show a significant difference between day and night in WT (Figure S16c,d). Similar to the light condition, in the dark, cytosolic NADPH levels in fully expanded leaves of 21‐day‐old *cynadp‐icdh* and *np‐gapdh* mutants were both lower than that of WT (Figure 4b), suggesting that AtcyNADP‐ICDH and Atnp‐GAPDH generate substantial cytosolic NADPH in the night.

## 4 Discussion

In Arabidopsis, cytosolic NADPH primarily originates from four NADP^+^‐reducing pathways: AtNADP‐MEs, cOPPP, AtcyNADP‐ICDH, and Atnp‐GAPDH. However, the relative contributions of the four pathways in different tissues remained elusive. In this study, the functions of these four pathways in Arabidopsis seedlings and leaves were investigated and clarified using the GUS reporter system, enzyme activity assays, and a second‐generation genetically encoded NADPH biosensor mCherry‐iNAP1 (Lim et al. 2024).

### 4.1 Cytosolic NADPH Metabolism in Shoots and Roots of 5‐Day‐Old Arabidopsis Seedlings

In 5‐day‐old WT plants, there was no significant day‐night difference in cytosolic NADPH levels, but the NADPH levels in the roots were remarkably higher than those in the shoots (Figure 1a–c). GUS experiments were performed to compare the promoter activities of the same gene in different tissues of the same seedlings/plants. GUS promoter analysis showed that the transcription levels of *AtNADP‐ME1‐2*, *AtG6PD5‐6*, *AtPGD1‐3*, and *AtcyNADP‐ICDH* were relatively higher in the roots than in the shoots (Figures S2–S10), while the expression of *Atnp‐GAPDH* was lower in the roots than in the shoots in 5‐day‐old seedlings (Figure S11). Our data indicated that AtNADP‐ME2 primarily provides a significant amount of cytosolic NADPH in roots but not to root tips nor shoots of 5‐day‐old plants (Figure 2b). This could be due to a higher transcript expression level of *AtNADP‐ME2* in the roots than in the shoots of 5‐day‐old plants (Figure S3c), and the content of its substrate, malate, in shoots of WT plants was only 0.72 times of that in roots (Krapp et al. 2011). *AtNADP‐ME2* was not expressed in root tips and therefore could not supply ample cytosolic NADPH in root tips (Figure S3c). The absence of AtNADP‐ME1 has no significant effect on cytosolic NADPH levels in roots and shoots, owing to its low catalytic efficiency (*K*_*cat*_ */K*_*mNADP*_ = 0.19 μM^−1^·s^−1^), which is markedly lower than that of AtNADP‐ME2 (*K*_*cat*_ */K*_*mNADP*_ = 4.5 μM^−1^·s^−1^) (Wheeler et al. 2005). Owing to the weak transcript expression levels of *AtNADP‐ME3* in 5‐day‐old seedlings (Figure S4c), its depletion exerted no discernible effect on cytosolic NADPH levels in 5‐day‐old seedlings despite its high catalytic efficiency (*K*_*cat*_ */K*_*mNADP*_ = 41.2 μM^−1^·s^−1^) (Table S2) (Wheeler et al. 2005).

As indicated by mCherry‐iNAP1, cOPPP serves as a source of cytosolic NADPH for both shoots and roots of 5‐day‐old seedlings (Figure 2c–e, Figure S13c,d). Notably, the transcript expression levels of *AtG6PD5‐6* and *AtPGD1‐3* were higher in the roots than in the shoots of 5‐day‐old plants (Figures S5–S9). Furthermore, the G6P content in the shoots was only 0.06 times of that in the roots (Krapp et al. 2011), suggesting that cOPPP could produce more NADPH in the roots than in the shoots of 5‐day‐old plants. AtcyNADP‐ICDH can provide cytosolic NADPH to both roots and shoots of 5‐day‐old seedlings (Figure 2f), as the isocitrate contents in roots and shoots were comparable (Krapp et al. 2011), and *AtcyNADP‐ICDH* was remarkably expressed in both roots and shoots of 5‐day‐old seedlings (Figure S10). Cotyledons of 5‐day‐old WT plants can efficiently perform photosynthesis (Shi et al. 2020). Therefore, the Calvin‐Benson cycle (CBC) can provide GAP to the shoots in the day, and glycolysis can supply GAP to the shoots at night and to the roots throughout the day in 5‐day‐old seedings. Although in 5‐day‐old plants Atnp‐GAPDH was robustly expressed in shoots and weakly expressed in roots (Figure S11c), the abundant GAP supply in shoots and roots throughout the day enables Atnp‐GAPDH to provide cytosolic NADPH to both shoots and roots (Figure 2g).

Cytosolic NADPH often serves as an electron donor for numerous essential cytosolic pathways in plant cells. For instance, ROS metabolism is an important pathway for cytosolic NADPH consumption. During ROS production, the transmembrane protein RboH facilitates the transfer of electrons from cytosolic NADPH to extracellular O_2_ to produce superoxide (O_2_^•^^−^), which is then rapidly converted to hydrogen peroxide (H_2_O_2_) by superoxide dismutase (Foyer and Noctor 2020; Kaur and Pati 2016; Su et al. 2024). The extracellular H_2_O_2_ can be transported into the cytosol via aquaporins and detoxified within the cytosol through the AsA‐GSH cycle and peroxidases (Prxs) (Dietz 2011; Mittler and Jones 2024; Noctor et al. 2012). In the cytosolic AsA‐GSH cycle, AtGR1 catalyzes the reduction of oxidized glutathione to reduced glutathione using NADPH as a reducing equivalent, and H_2_O_2_ oxidizes AsA to monodehydroascorbic acid through ascorbate peroxidase (Noctor et al. 2012; Noctor et al. 2000). In addition, cytosolic H_2_O_2_ can also be reduced to water by Prx_red_, and the generated Prx_ox_ is reduced to Prx_red_ by reduced thioredoxin (Trx_red_). The resulting Trx_ox_ is then converted back to Trx_red_ by AtNTRA with electrons derived from cytosolic NADPH (Jedelská et al. 2020). Under stress conditions, such as hypoxia and salt stress, elevated cytosolic Ca^2+^ levels can activate AtNDB1 to consume cytosolic NADPH, thereby mitigating ROS production (Rasmusson et al. 2020; Sweetman et al. 2019). Among key genes participated in ROS metabolism, transcript expression levels of *AtRboHB‐C* and *AtRbohF* were considerably elevated in roots compared with the whole WT seedlings (Table S1), which demonstrated that the high cytosolic NADPH levels in roots may serve as substrates for AtRbohC and AtRbohF, to negatively control hydrotropic growth and promote primary root growth, respectively (Krieger et al. 2016; Kwak et al. 2003).

On the other hand, the elongation of acyl‐CoA into VLCFA by the FAE complex anchored to the cytosolic side of the ER membrane is also a major pathway for consuming cytosolic NADPH. VLCFAs account for 29%–36% of the total acyl chains in roots, while account for only 5% of the total acyl chains in leaves (Batsale et al. 2021). This is because VLCFAs are precursors of cuticular waxes and suberin, which are essential for Casparian strip formation in roots (Batsale et al. 2021). The FAE complex subunit AtKCR1 and AtG6PD5.4 co‐localize on the ER membrane, and AtG6PD5.4 can facilitate the anchoring of AtPGD2 and AtPGL2 to the ER membrane, thereby supporting the operation of ER membrane‐localized cOPPP (Linnenbrügger et al. 2022). The *in planta* observations revealed that the *g6pd5.4/6* mutant exhibited decreased NADPH content in roots and shoots compared with WT (Figure 2c). This finding is consistent with previous findings of reduced NADPH levels and VLCFA contents, and shorter root meristem length in the *g6pd5/6* mutant line, indicating that NADPH supply by cOPPP is essential for VLCFA biosynthesis (Li et al. 2025; Linnenbrügger et al. 2022; Ruan et al. 2022). The mRNA expression level of *AtKCR1* and genes in cOPPP (*AtG6PD5‐6* and *AtPGD1‐3*) in shoot was lower than in roots (Table S1) (Qin et al. 2009), and cOPPP could be more active in roots than in shoots, as discussed above. These findings indicate that high levels of cytosolic NADPH in roots can function as substrates for FAE to promote VLCFA synthesis and sustain root growth in 5‐day‐old seedings. Collectively, all the findings further support our *in planta* observation of higher cytosolic NADPH in the roots compared with shoots.

### 4.2 Cytosolic NADPH Metabolism in Leaves Differs Under Light and Dark Conditions

In rosette leaves of 21‐day‐old WT plants, cytosolic NADPH levels were significantly lower at the end of the day than at night (Figure 1e–f). Promoter‐GUS analysis showed that GUS staining was intense in the fully expanded leaves of 21‐day‐old plants of *AtNADP‐ME2*, *AtPGD2*, *AtcyNADP‐ICDH*, and *Atnp‐GAPDH*, but the staining was weak in the fully expanded leaves of *AtNADP‐ME1*, *AtNADP‐ME3*, *AtG6PD5*, *AtG6PD6, AtPGD1*, and *AtPGD3* (Figures S2–S11).

In the day, malate acts as a vehicle for transporting the excess reductants derived from both chloroplast photosynthesis and mitochondrial process of photorespiration to the cytosol (Lim et al. 2020). At the same time, light activates plasma membrane H^+^‐ATPase (AHA) to pump protons out of the cell, causing cytosolic alkalinization, thereby promoting the transport of malate in the cytosol to the vacuole through the vacuolar malate/citrate antiporter AttDT against the concentration gradient (Elsässer et al. 2020; Frei et al. 2018; Hoffmann et al. 2020). Thus, malate accumulates continuously in vacuole during the day in leaves. In spinach (*Spinacia oleracea*), by the end of the day, the malate concentrations in the vacuole were 29 times higher than that in the cytosol (Table S3) (Gerhardt et al. 1987). Accordingly, at the end of the day, the malate content in cytosol of Arabidopsis leaves was approximately 193 μM, which was much lower than the *K*_m malate_ value, 3.33 mM, of AtNADP‐ME2 (Tables S2–S3) (Annunziata et al. 2018; Wheeler et al. 2005). Although *AtNADP‐ME2* was highly expressed in leaves (Table S4) (Jung et al. 2024) and AtNADP‐ME2 contributed most to the major global NADP‐ME activities (Wheeler et al. 2021; Wheeler et al. 2005), it may not produce a significant amount of NADPH during the day due to low availability of its substrate in the cytosol (Figure S14b). The mRNA expression of *AtNADP‐ME1* and *AtNADP‐ME3* in leaves was quite low and limited (Figures S2, S4, and Table S1), and AtNADP‐ME1 and AtNADP‐ME3 contribute little to the major global NADP‐ME activities in WT leaves (Wheeler et al. 2005), so these findings could explain why they are not the main cytosolic NADPH sources in leaves (Figure S14 and Figure 4).

During the day, the substantial amount of NADH produced by photorespiration in mitochondria suppresses the activity of mitochondrial pyruvate dehydrogenase complex (mtPDC), mitochondrial NAD‐dependent isocitrate dehydrogenase (mtNAD‐ICDH), and oxoglutarate dehydrogenase complex (OGDH) in the tricarboxylic acid (TCA) cycle. Therefore, the TCA cycle during the day is not a cycle and cannot transport large amounts of citrate to cytosol as it does in the night (Gardeström and Igamberdiev 2016; Sweetlove et al. 2010). Meanwhile, the vacuole releases a great deal of citrate stored overnight into the cytosol through AttDT (Elsässer et al. 2020; Frei et al. 2018; Hoffmann et al. 2020). As the aconitase (ACO)‐catalyzed conversion of citrate to isocitrate is reversible (0.22 kcal/mol, biocyc.org/) and only 6% isocitrate is produced at equilibrium (91% of the product is citrate and 3% is cis‐aconitate), the intracellular concentration of isocitrate is remarkably lower than that of citrate (Rahikainen et al. 2025). At the end of the day, the content of isocitrate (32 μM) in Arabidopsis leaves was close to the *K*_m isocitrate_ value (42 μM) of the AtcyNADP‐ICDH enzyme (Tables S2–S3) (Annunziata et al. 2018; Leterrier et al. 2012). Furthermore, the mRNA expression level of *AtNADP‐ICDH* in leaves was high in the day (Figure S10 and Table S4), and AtcyNADP‐ICDH contributed to considerable NADP‐ICDH activity in WT leaves in light (Figure S16c) (Leterrier et al. 2012). All of this evidence further supports our observation where the AtcyNADP‐ICDH can produce a large amount of cytosolic NADPH in the day (Figure 3b). During photosynthesis, the ample GAP generated via the CBC is released to the cytosol. Some of the cytosolic GAP can be used for cytosolic NADPH production by Atnp‐GAPDH, and the other is involved in sucrose synthesis (Lu et al. 2025; Noronha et al. 2018). The mRNA expression levels of *Atnp‐GAPDH* in leaves were high (Figure S11 and Table S4), the np‐GAPDH activity in WT leaves was high in the light (Figure S16d), and GAP is abundantly supplied during the day, so Atnp‐GAPDH can provide a large amount of cytosolic NADPH in the day in leaves (Figure 3c). In addition to supplying non‐photosynthetic tissues, sucrose synthesized during the day is stored in the vacuole (Noronha et al. 2018). G6P, a key intermediate in sucrose synthesis, accumulated continuously in cytosol. Sucrose also accumulated in cytosol and vacuole steadily during photosynthesis and its levels in both compartments reached their peak levels at the end of the day (Gerhardt et al. 1987). Therefore, the ample supply of cytosolic G6P during the day can drive the cOPPP to supply abundant NADPH in cytosol in leaves (Figure 3a).

During the night, photosynthesis ceases, and the glycolysis can generate substantial G6P in chloroplasts and cytosol (Fernie et al. 2004). In the night, the loss of individual cytosolic G6PD or PGD isoform impaired cytosolic NADPH levels in leaves (Figure 4), while only the leaves of *g6pd5.4/6* mutant showed significantly reduced cytosolic NADPH levels in the day compared with WT (Figure 3a and Figure S15), indicating that the cOPPP can generate more NADPH at night than in the day. In the day, due to the functional redundancy of cytosolic G6PD or PGD isoenzymes (Andriotis and Smith 2019; Lutterbey and von Schaewen 2016; Wakao et al. 2008), the loss of a single G6PD or PGD isoform has no significant effect on cOPPP function and leaf NADPH levels (Figure S15). Since there was no difference in cytosolic G6PDH activity between day and night in WT leaves (Figure S16b), the G6PDH activity was not the primary factor resulting higher NADPH supply from cOPPP at night than during the day. This difference could be attributed to the supply of G6P at night is significantly higher than that during the day. Taking spinach as an example, spinach leaves consume approximately 14 mM sucrose at night (Table S3). Since each molecule of sucrose is degraded and phosphorylated to produce one molecule of G6P and one molecule of fructose 6‐phosphate (F6P), the levels of G6P released into the cytosol of spinach leaves at night were approximately 17‐fold greater than those at the end of the day (Table S3). In addition to cOPPP, G6P can be consumed in glycolysis to generate GAP, which can be utilized by Atnp‐GAPDH to produce NADPH, and can also continue to enter the glycolysis to generate NADH (Fernie et al. 2004; Piattoni et al. 2010; Rius et al. 2006). The active glycolysis at night can produce a large amount of GAP. The transcript expression level of *Atnp‐GAPDH* at night was 1.79 times greater than that in the day (Table S4), and np‐GAPDH activity was comparable in WT leaves in both light and dark conditions (Figure S16d). These findings support that Atnp‐GAPDH can produce a large amount of NADPH at night (Figure 4).

Meanwhile, the acidification of cytosol prompts vacuole to release malate accumulated during the day to the cytosol through AttDT (Elsässer et al. 2020; Frei et al. 2018; Hoffmann et al. 2020). In Arabidopsis leaves, the content of malate released from vacuole into the cytosol at night was approximately 3.8 mM, 1.14‐fold the *K*_m malate_ value of AtNADP‐ME2 (Tables S2–S3). Accordingly, although the mRNA expression level of *AtNADP‐ME2* at night was only 0.43 times that of the day (Table S4), and the cytosolic NADP‐ME activity was comparable in WT leaves in light and dark conditions (Figure S16a), AtNADP‐ME2 can efficiently produce large amounts of NADPH at night (Figure 4). The nocturnal cessation of photorespiration allows the TCA cycle to proceed normally, facilitating substantial citrate export to the cytosol. Subsequently, the cytosolic citrate is predominantly transported into the vacuole for storage via AttDT (Elsässer et al. 2020; Frei et al. 2018; Hoffmann et al. 2020). Due to the limited efficiency of ACO, leaf isocitrate concentration remained relatively stable throughout the day, which was 36 μM in the night and near the *K*_m isocitrate_ value of AtcyNADP‐ICDH (Tables S2–S3). In addition, the cytosolic NADP‐ICDH activity was comparable in WT leaves in both light and dark conditions (Figure S16c). Hence, AtcyNADP‐ICDH can efficiently catalyze the conversion of isocitrate to 2‐OG and supply NADPH at night (Figure 4). Taken together, cOPPP and Atnp‐GAPDH are essential not only for NADPH production but also for the regulation of carbon metabolic flux. Therefore, in this study, the delayed rosette growth of *g6pd5.4/6* and *np‐gapdh* mutants may be due to impaired carbon metabolism (Figure S1) (Rius et al. 2006; Ruan et al. 2022).

Unlike seedlings, cytosolic NADPH consumption pathways in leaves differ between day and night. During the day, the bypass of photorespiration catalyzing the conversion of hydroxypyruvate to glycerate in the cytosol can consume cytosolic NADPH via AtHPR2, as inhibition of photorespiration with aminoacetonitrile increased cytosolic NADPH levels in the cytosol under light (Lim et al. 2020; Timm et al. 2008). In addition, cytosolic NADPH can also be used for the detoxification of ROS and the biosynthesis of flavonoid precursors. In leaves, H_2_O_2_ levels oscillate under circadian control, with daytime production primarily driven by photosynthesis and photorespiration, shifting to respiratory pathways at night (Lai et al. 2012). The H_2_O_2_ levels at the end of the day were lower than those in the middle of the night in WT leaves (Lai et al. 2012). In the night, the mRNA expression of *AtGR1* was 1.81‐fold higher than during the day (Table S4), and the elevated cytosolic NADPH levels at night could serve as a substrate for AtGR1 to drive AsA‐GSH cycle and ROS detoxification. The mRNA expression levels of *AtATR1* exhibited a 2.14‐fold nocturnal increase (Table S4), paralleling the rhythmicity of its downstream cytochrome P450 genes. Cytochrome P450 members C4H (encoded by *AtCYP73A5*), anchored to the cytosolic side of the ER membrane, and flavonoid 3'‐monooxygenase (F3H, encoded by *AtCYP75B1*), localized to cytosol, are involved in the phenylpropanoid pathway and its downstream flavonoid pathway, respectively. In WT leaves, the transcript expression levels of *AtCYP73A5* and *AtCYP75B1* were roughly 6‐times and 15‐times higher at night than at the end of the day, respectively (Pan et al. 2009). These findings suggest that AtATR1 can consume substantial cytosolic NADPH at night and transfer electrons to C4H and F3H, thereby driving flavonoid precursors biosynthesis. It has been reported previously that AtATR1 colocalizes with AtG6PD5.4 on the ER membrane (Linnenbrügger et al. 2022), illustrating that cOPPP may act as a key NADPH source for AtATR1, and the active cOPPP in the night can provide a large amount of NADPH for AtATR1.

In summary, in 5‐day‐old Arabidopsis plants, AtNADP‐ME2 provides cytosolic NADPH in the roots but not in root tips, cOPPP can supply more cytosolic NADPH in the root than in the shoot, and AtcyNADP‐ICDH and Atnp‐GAPDH can provide cytosolic NADPH in both the shoot and root (Figure 2a). The 5‐day‐old Arabidopsis seedlings are in a fast‐grown stage, and the high cytosolic NADPH levels in roots can serve as a substrate for RboH and support the synthesis of VLCFAs, thereby promoting root growth development. A schematic diagram is presented here to illustrate the diurnal differences in NADPH supply in the leaves (Figure 5). In leaves, AtNADP‐ME2 mainly functions at night, cOPPP generates more cytosolic NADPH at night than during the day, and AtcyNADP‐ICDH and Atnp‐GAPDH function in both day and night (Figure 5). The high levels of cytosolic NADPH at night can be used for ROS metabolism and biosynthesis of flavonoid precursors (Figure 5). The efficient photorespiration under illumination can consume significant amounts of cytosolic NADPH via AtHPR2, exacerbating the diurnal and nocturnal differences in cytosolic NADPH levels (Figure 5). Overall, this study elucidated the contribution of various NADPH supply pathways in Arabidopsis seedlings and leaves under normal growth conditions. Given the important functions of NADP‐ME2 (Wu et al. 2022), OPPP (de Freitas‐Silva et al. 2017; Landi et al. 2024), cyNADP‐ICDH (Leterrier et al. 2012; Leterrier et al. 2007), and np‐GAPDH (Mhamdi and Noctor 2016) in stress responses, their contribution to NADPH supply under stress conditions can be further explored in the future.

**FIGURE 5:**
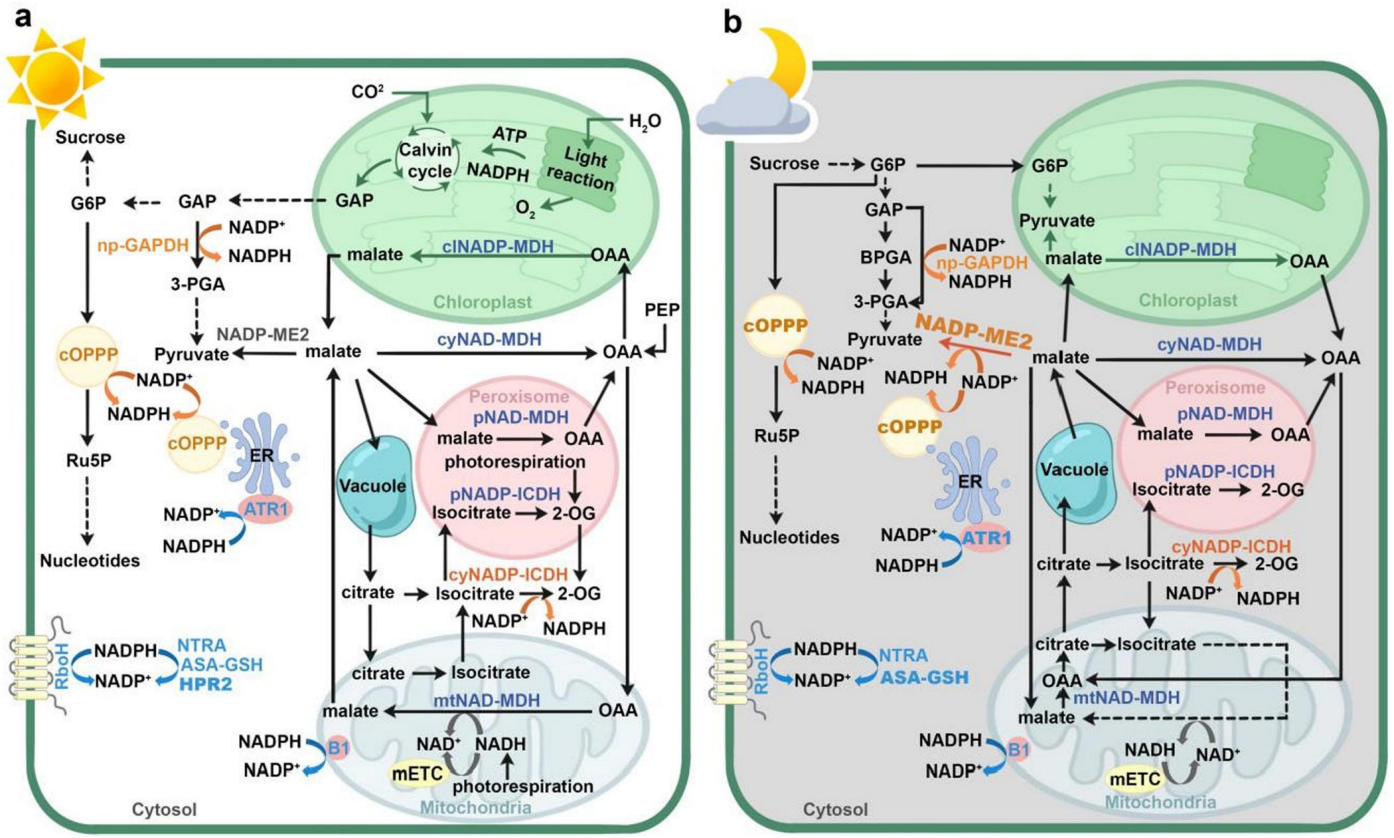
Cytosolic NADPH metabolism in the leaves of Col‐0 Arabidopsis. (a) During the day, cOPPP, cyNADP‐ICDH, and np‐GAPDH supply cytosolic NADPH to leaves. Meanwhile, the cytosolic bypass catalyzing the conversion of hydroxypyruvate to glycerate in photorespiration can consume large amounts of NADPH via AtHPR2. (b) At night, NADP‐ME2 utilizes malate accumulated during the day to generate large amounts of cytosolic NADPH. The cOPPP pathway can produce more NADPH at night. In addition, cyNADP‐ICDH and np‐GAPDH can also supply cytosolic NADPH at night. High levels of cytosolic NADPH can serve as a substrate for AtGR1 to participate in the cytosolic AsA‐GSH cycle and ROS detoxification, and it can also drive hydroxylation reactions that produce precursors for flavonoids through AtATR1. The NADPH‐producing enzymes are indicated in orange, and the NADPH‐consuming enzymes are shown in blue. Pathways that are enhanced in the day or night are indicated by shaded text. ASA‐GSH, Ascorbate‐Glutathione cycle; ATP, Adenosine Triphosphate; ATR, cytochrome P450 reductase; BPGA, 1,3‐Bisphosphoglycerate; cyNAD‐MDH, cytosolic NAD‐dependent malate dehydrogenase; clNADP‐MDH, chloroplast NADP‐dependent malate dehydrogenase; cyNADP‐ICDH, cytosolic NADP‐dependent isocitrate dehydrogenase; ER, endoplasmic reticulum; G6P, Glucose 6‐phosphate; GAP, glyceraldehyde 3‐phosphate; HPR2, hydroxypyruvate reductase 2; mETC, mitochondrial electron transport chain; mtNAD‐MDH, mitochondrial NAD‐dependent malate dehydrogenase; np‐GAPDH, non‐phosphorylating glyceraldehyde 3‐phosphate dehydrogenase; NADP‐ME, NADP‐dependent malate enzyme; NDB1 (B1), alternative NADPH dehydrogenase B1; NTRA, NADPH‐dependent thioredoxin reductase A; OAA, oxaloacetate; OPPP, oxidative pentose phosphate pathway; PEP, phosphoenolpyruvate; pNAD‐MDH, peroxisomal NAD‐dependent malate dehydrogenase; pNADP‐ICDH, peroxisomal NADP‐dependent isocitrate dehydrogenase; RboH, respiratory burst oxidase homologs; TCA cycle, tricarboxylic acid cycle; 3‐PGA, 3‐phosphoglycerate; 2‐OG; 2‐oxoglutarate. The cOPPP anchored to the cytosolic face of the ER membrane is constituted of G6PD5.4 (glucose‐6‐phosphate dehydrogenase), 6‐phosphogluconolactonase (PGL), and PGD2 (6‐phosphogluconate dehydrogenase); the cOPPP in the cytosol is constituted of G6PD5/6, PGL, and PGD1‐3.

## Conflicts of Interest

The authors declare no conflicts of interest.

## Supporting information


Supporting File

## Data Availability

All study data were included in the article and Supporting Information.
