# Supplementary material for: Spatiotemporal Dynamics of Cytosolic NADPH in Living Arabidopsis thaliana
Source: Plant Cell Environ. 2026 Aug 11;49(11):9101–16. doi: 10.1111/pce.70806 (PMC13646676; doi:10.1111/pce.70806)
Supplement: Supplementary file 1 [file PCE-49-9101-s001.pdf]

Supporting Information

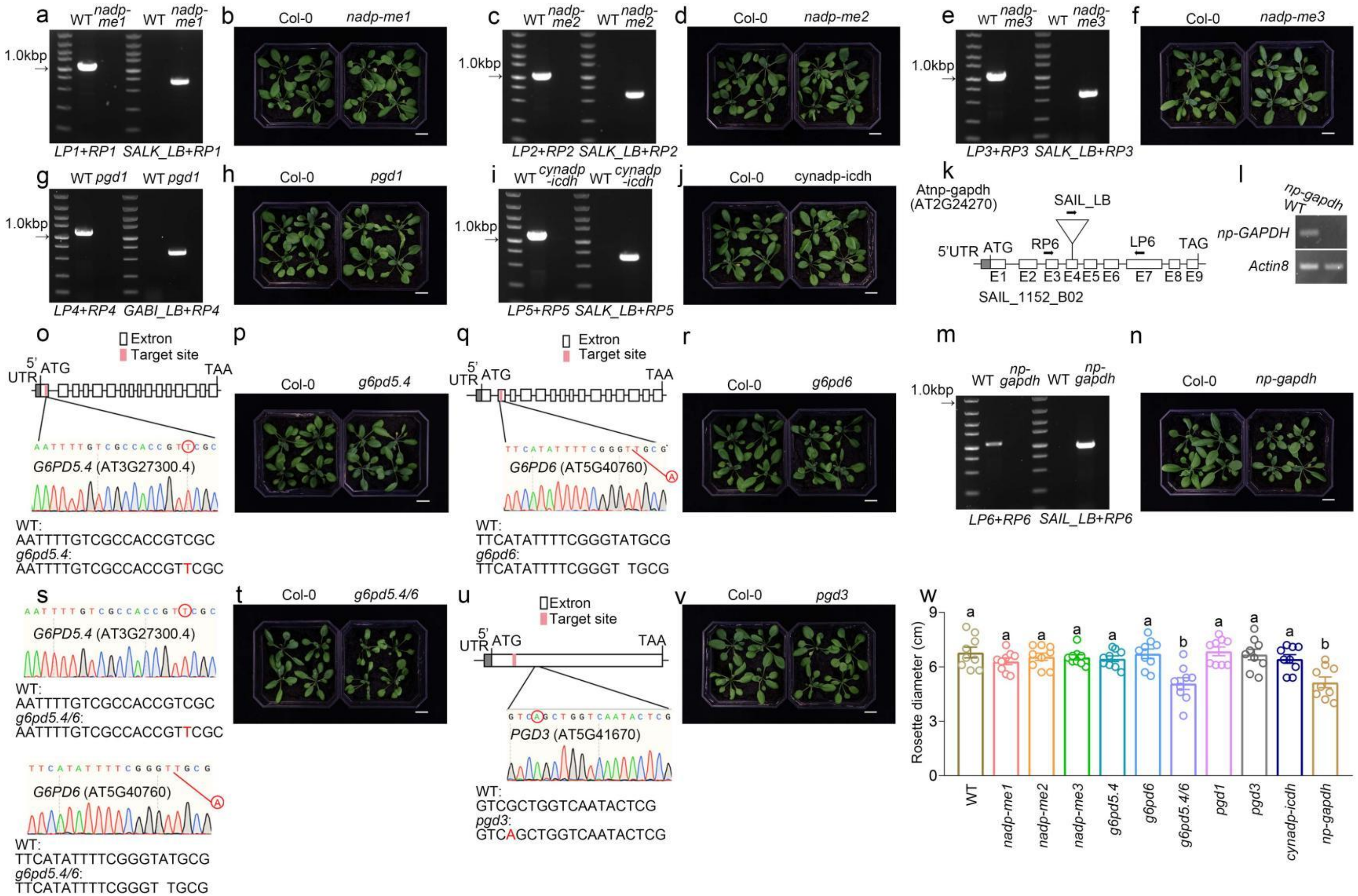

Figure S1. Confirmation and phenotypes of mutants used in this study.

Genotyping analysis of **(a)** *nadp-me1* (SALK-036898), **(c)** *nadp-me2* (SALK-020607), **(e)** *nadp-me3* (SALK-139336), **(g)** *pgd1* (GABI_762C06), **(i)** *cynadp-icdh* (SALK-056247), and **(m)** *np-gapdh* (SAIL_1152_B02) mutants. **(k)** Schematic representation of T-DNA insertion site of *np-gapdh* mutant. White boxes represent the gene exons. UTR, untranslated region. **(l)** Confirmation of *np-gapdh* knock-out by RT-PCR. The housekeeping gene *Actin8* was employed as control. Phenotypes of 30-day-old **(b)** *nadp-me1*, **(d)** *nadp-me2*, **(f)** *nadp-me3*, **(h)** *pgd1*, **(j)** *cynadp-icdh*, **(n)** *np-gapdh*, **(p)** *g6pd5.4*, **(r)** *g6pd6*, **(t)** *g6pd5.4/6*, and **(v)** *pgd3* seedlings. sgRNA and schematic representation of the **(o)** *G6PD5.4* locus, **(q)** *G6PD6* locus for *g6pd5.4* mutant and *g6pd6* mutant, respectively. **(s)** Schematic representation of the *G6PD5.4* and *G6PD6* locus for *g6pd5.4/6* mutant. **(u)** Schematic representation of the *PGD3* locus for *pgd3* mutant. **(w)** A comparison of rosette diameters of 30-day-old plants of WT and mutants used in this study. Significant differences of rosette diameters were determined by Tukey's HSD ($p < 0.05$); $n = 9$ plants for each column; errors bar ± SEM. Scale bar in b, d, f, h, j, n, 1, p, r, t, and v, 1 cm.

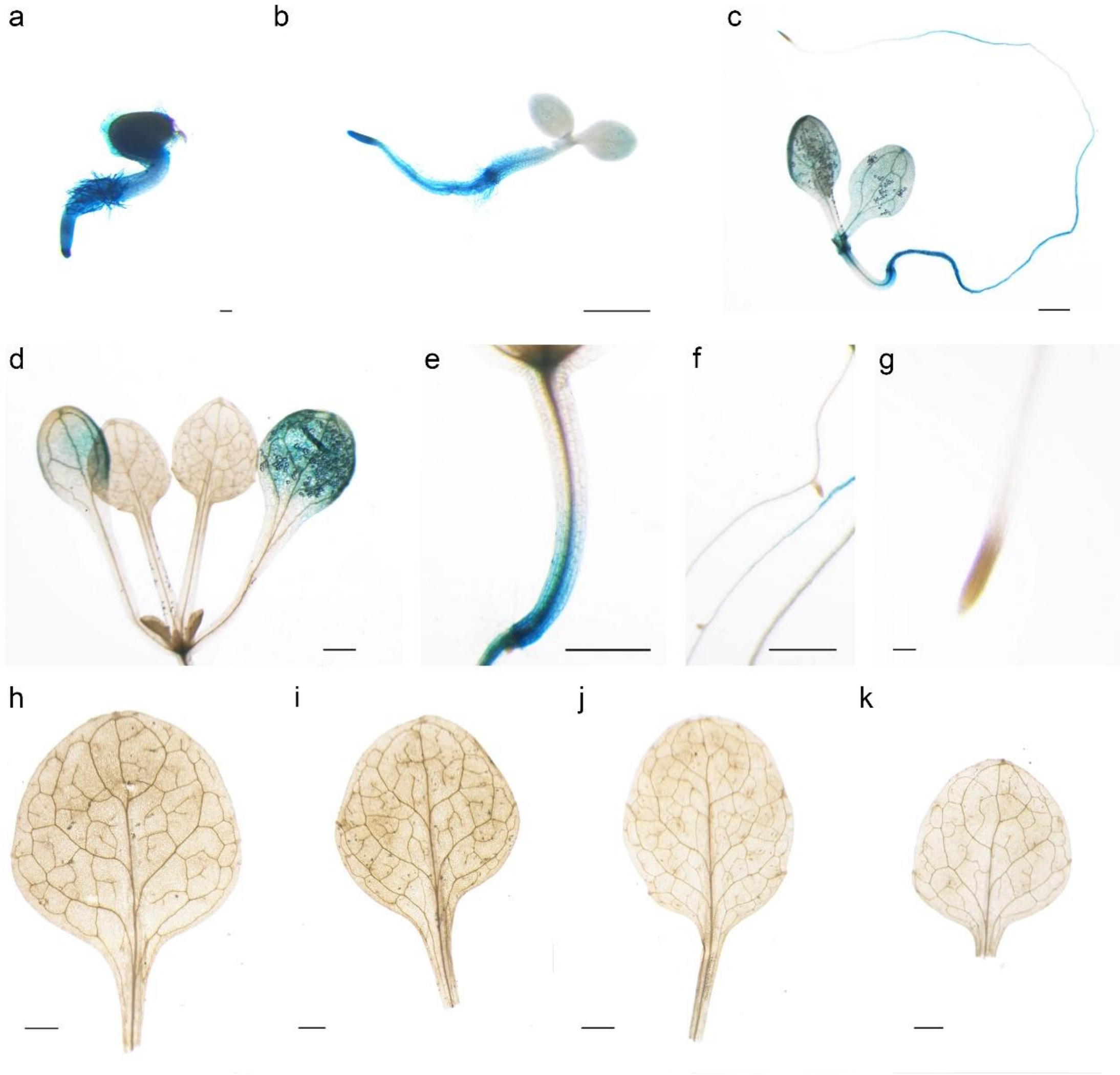

Figure S2. Histochemical GUS staining of *AtNADP-ME1::Pro-GUS* transgenic Arabidopsis seedlings.

Histochemical localization of GUS activity in **(a)** 1-day-old, **(b)** 2-day-old, **(c)** 5-day-old, **(d)** cotyledons, **(e)** transition zone, **(f)** roots, and **(g)** root tips of 10-day-old, and **(h-k)** leaves of 21-day-old *AtNADP-ME1::Pro-GUS* transgenic Arabidopsis plants. Leaves ordered from the oldest to the youngest (left to right).

Scale bar in a and g, 100 μm. Scale bar in b-f, h-k, 1000 μm.

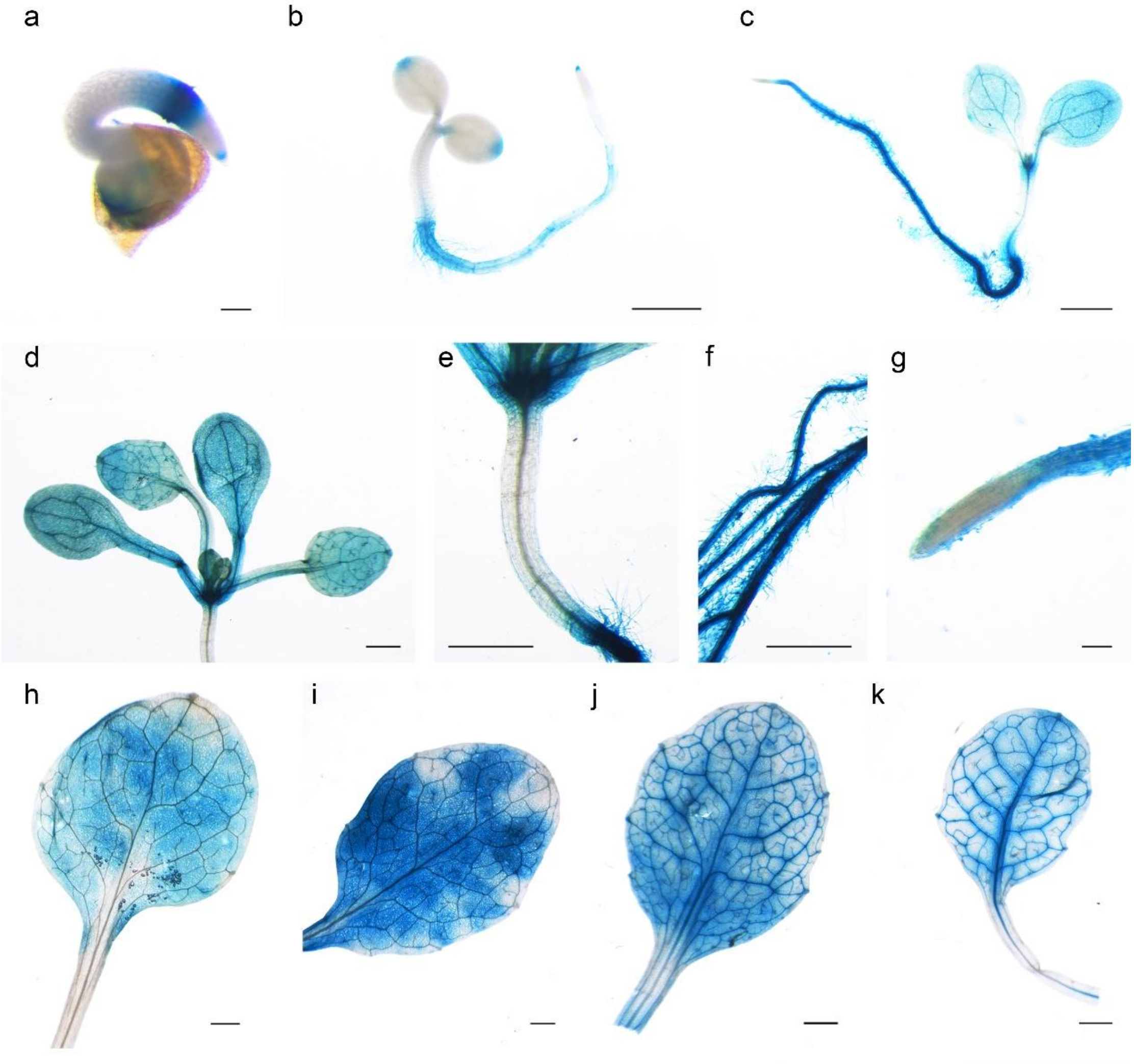

Figure S3. GUS histochemical staining in *AtNADP-ME2::Pro-GUS* transgenic Arabidopsis seedlings.

Histochemical localization of GUS activity in **(a)** 1-day-old, **(b)** 2-day-old, **(c)** 5-day-old, **(d)** cotyledon, **(e)** transition zone, **(f)** root, and **(g)** root tip of 10-day-old, and **(h-k)** Leaves of 21-day-old *AtNADP-ME2::Pro-GUS* transgenic Arabidopsis plants. Leaves ordered from the oldest to the youngest (left to right).

Scale bar in a and g, 100 μm. Scale bar in b-f, h-k, 1000 μm.

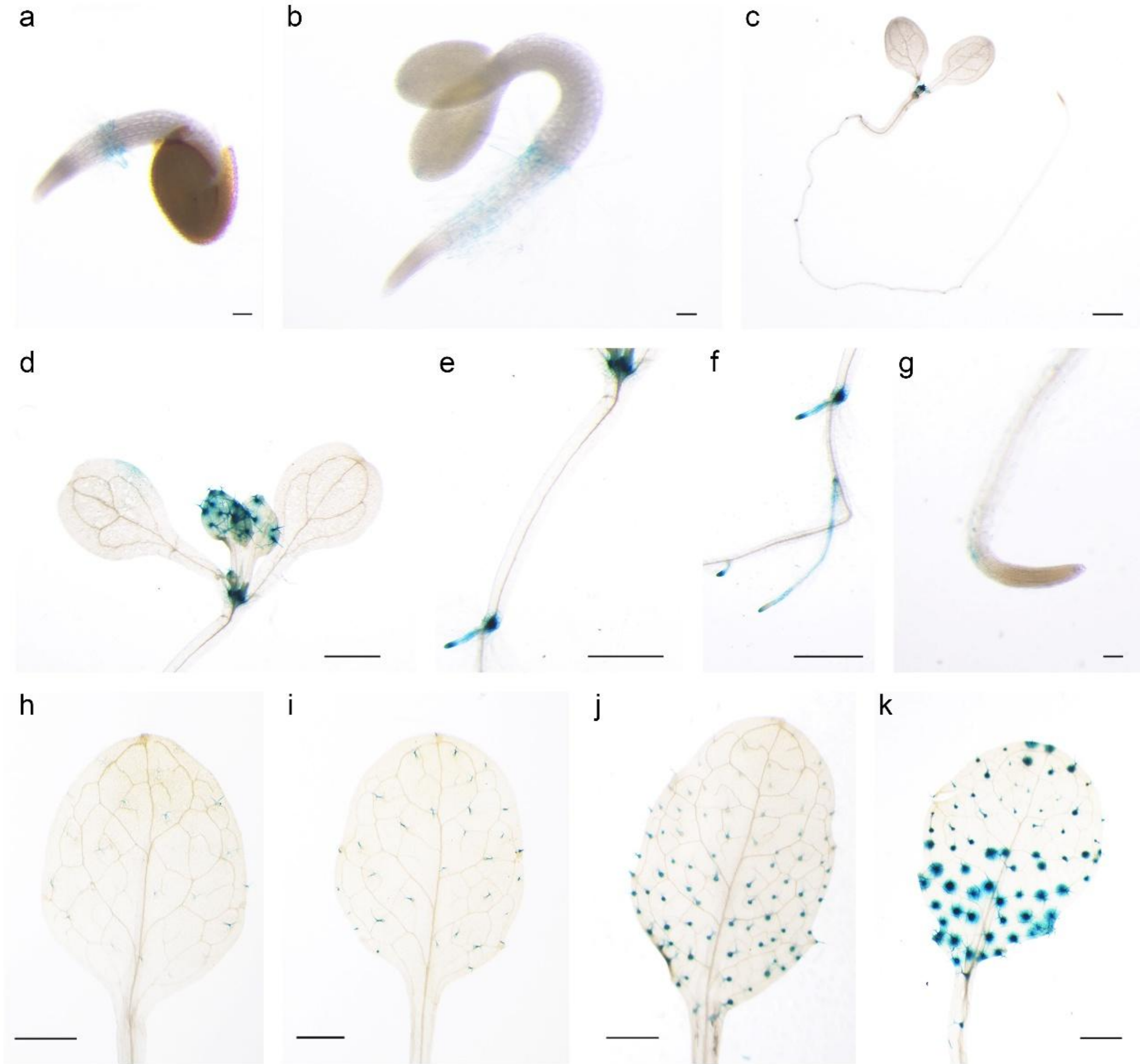

Figure S4. GUS histochemical staining in *AtNADP-ME3::Pro-GUS* transgenic Arabidopsis seedlings.

Histochemical localization of GUS activity in **(a)** 1-day-old, **(b)** 2-day-old, **(c)** 5-day-old, **(d)** cotyledon, **(e)** transition zone, **(f)** root, and **(g)** root tip of ten-day-old, and **(h-k)** Leaves of 21-day-old *AtNADP-ME3::Pro-GUS* transgenic Arabidopsis plants. Leaves ordered from the oldest to the youngest (left to right).

Scale bar in a, b, and, g 100 μm. Scale bar in c-f, h-k, 1000 μm.

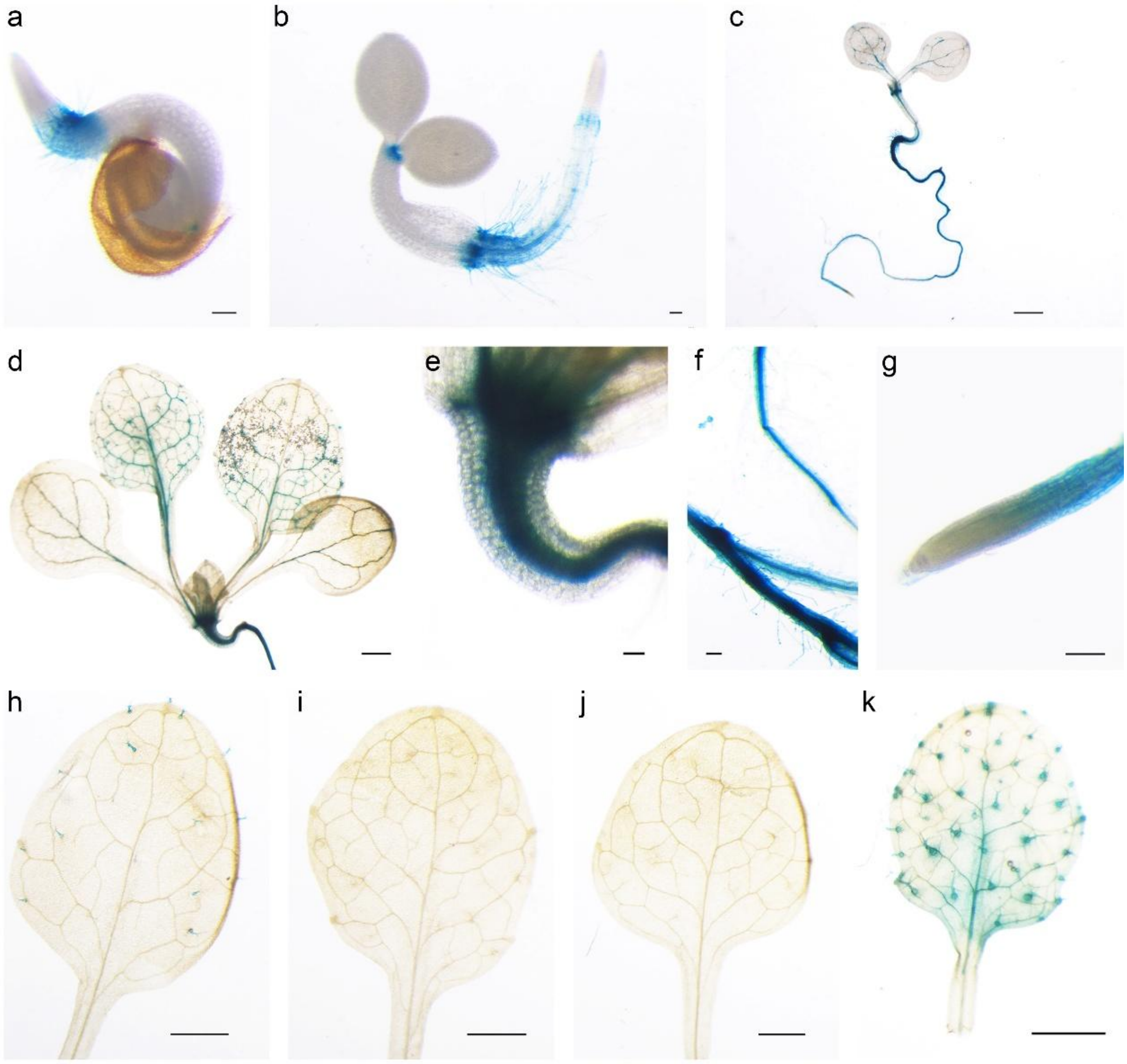

Figure S5. GUS histochemical staining in *AtG6PD5::Pro-GUS* transgenic Arabidopsis seedlings.

Histochemical localization of GUS activity in **(a)** 1-day-old, **(b)** 2-day-old, **(c)** 5-day-old, **(d)** cotyledon, **(e)** transition zone, **(f)** root, and **(g)** root tip of ten-day-old, and **(h-k)** Leaves of 21-day-old *AtG6PD5::Pro-GUS* transgenic Arabidopsis plants. Leaves ordered from the oldest to the youngest (left to right).

Scale bar in a, b, e, f and g 100µm. Scale bar in c, d, h, i, j, and k, 1000 µm.

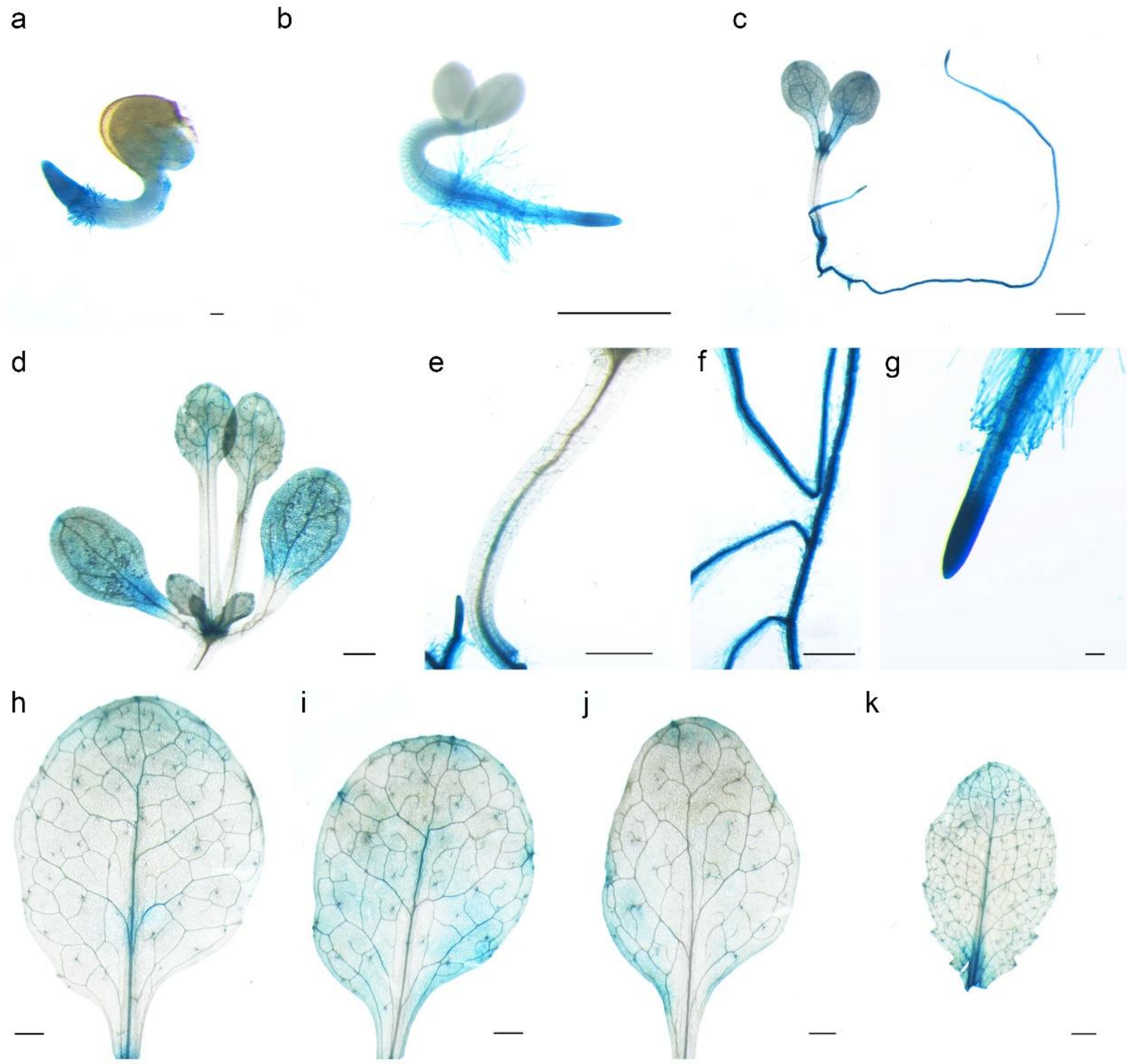

Figure S6. GUS histochemical staining in *AtG6PD6::Pro-GUS* transgenic Arabidopsis seedlings.

Histochemical localization of GUS activity in **(a)** 1-day-old, **(b)** 2-day-old, **(c)** 5-day-old, **(d)** cotyledon, **(e)** transition zone, **(f)** root, and **(g)** root tip of ten-day-old, and **(h-k)** Leaves of 21-day-old *AtG6PD6::Pro-GUS* transgenic Arabidopsis plants. Leaves ordered from the oldest to the youngest (left to right).

Scale bar in a, and g, 100µm. Scale bar in b-f, h-k 1000 µm.

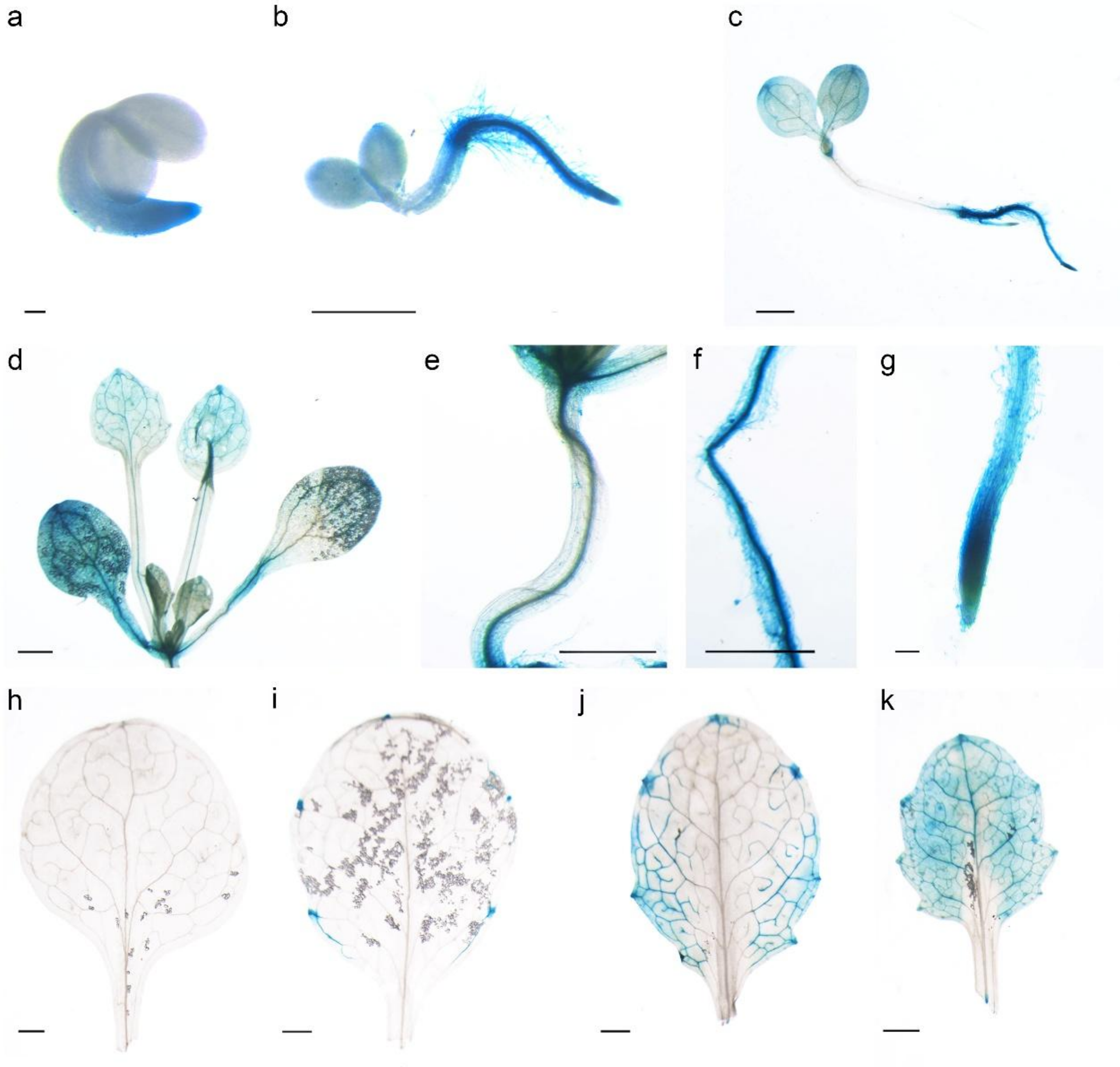

Figure S7. GUS histochemical staining in *AtPGD1::Pro-GUS* transgenic Arabidopsis seedlings.

Histochemical localization of GUS activity in **(a)** 1-day-old, **(b)** 2-day-old, **(c)** 5-day-old, **(d)** cotyledon, **(e)** transition zone, **(f)** root, and **(g)** root tip of ten-day-old, and **(h-k)** Leaves of 21-day-old *AtPGD1::Pro-GUS* transgenic Arabidopsis plants. Leaves ordered from the oldest to the youngest (left to right).

Scale bar in a, and g, 100μm. Scale bar in b-f, h-k 1000 μm.

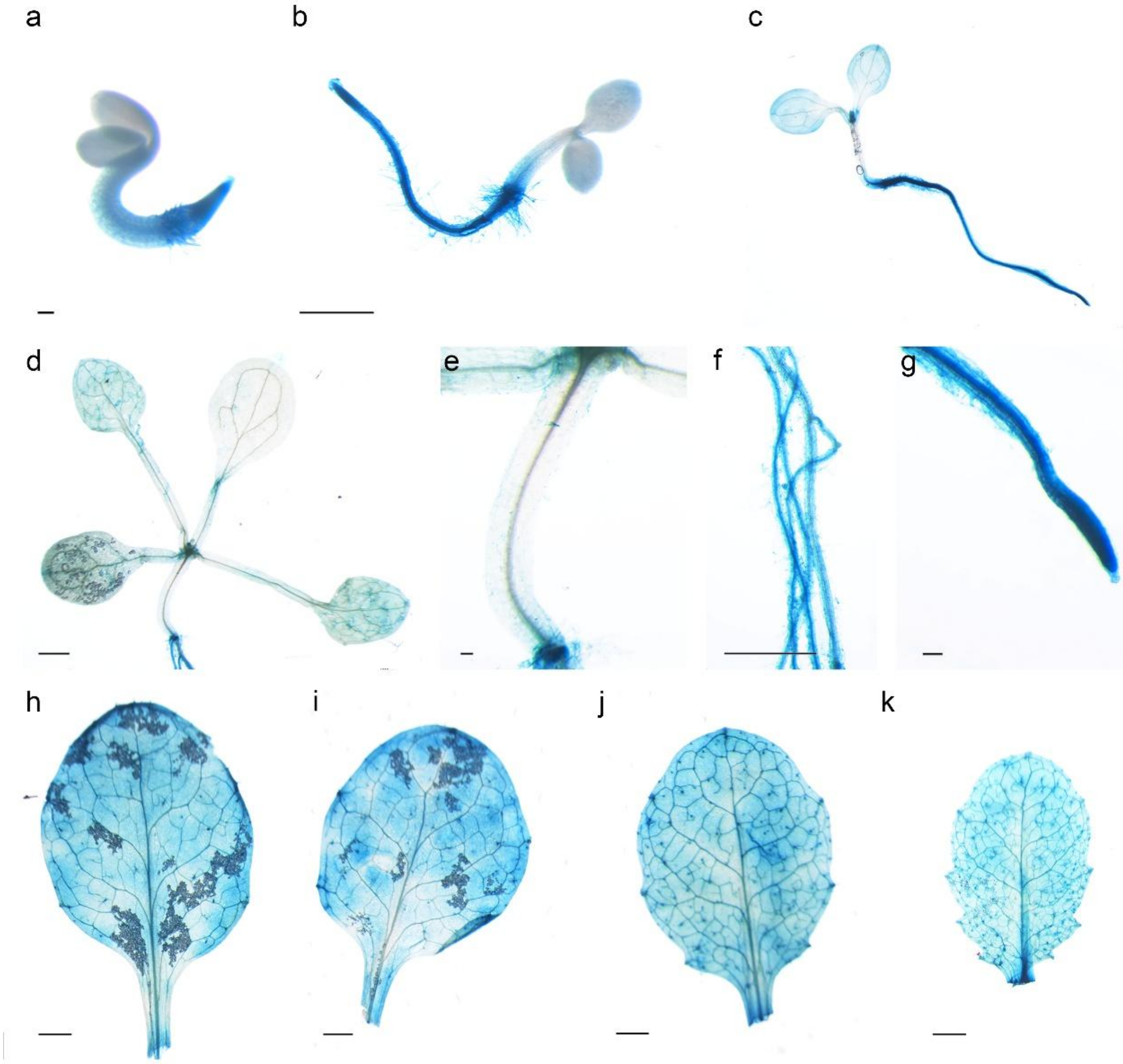

Figure S8. GUS histochemical staining in *AtPGD2::Pro-GUS* transgenic Arabidopsis seedlings.

Histochemical localization of GUS activity in **(a)** 1-day-old, **(b)** 2-day-old, **(c)** 5-day-old, **(d)** cotyledon, **(e)** transition zone, **(f)** root, and **(g)** root tip of 10-day-old, and **(h-k)** Leaves of 21-day-old *AtPGD2::Pro-GUS* transgenic Arabidopsis plants. Leaves ordered from the oldest to the youngest (left to right).

Scale bar in a, e and g, 100μm. Scale bar in b-d, f, h-k 1000 μm.

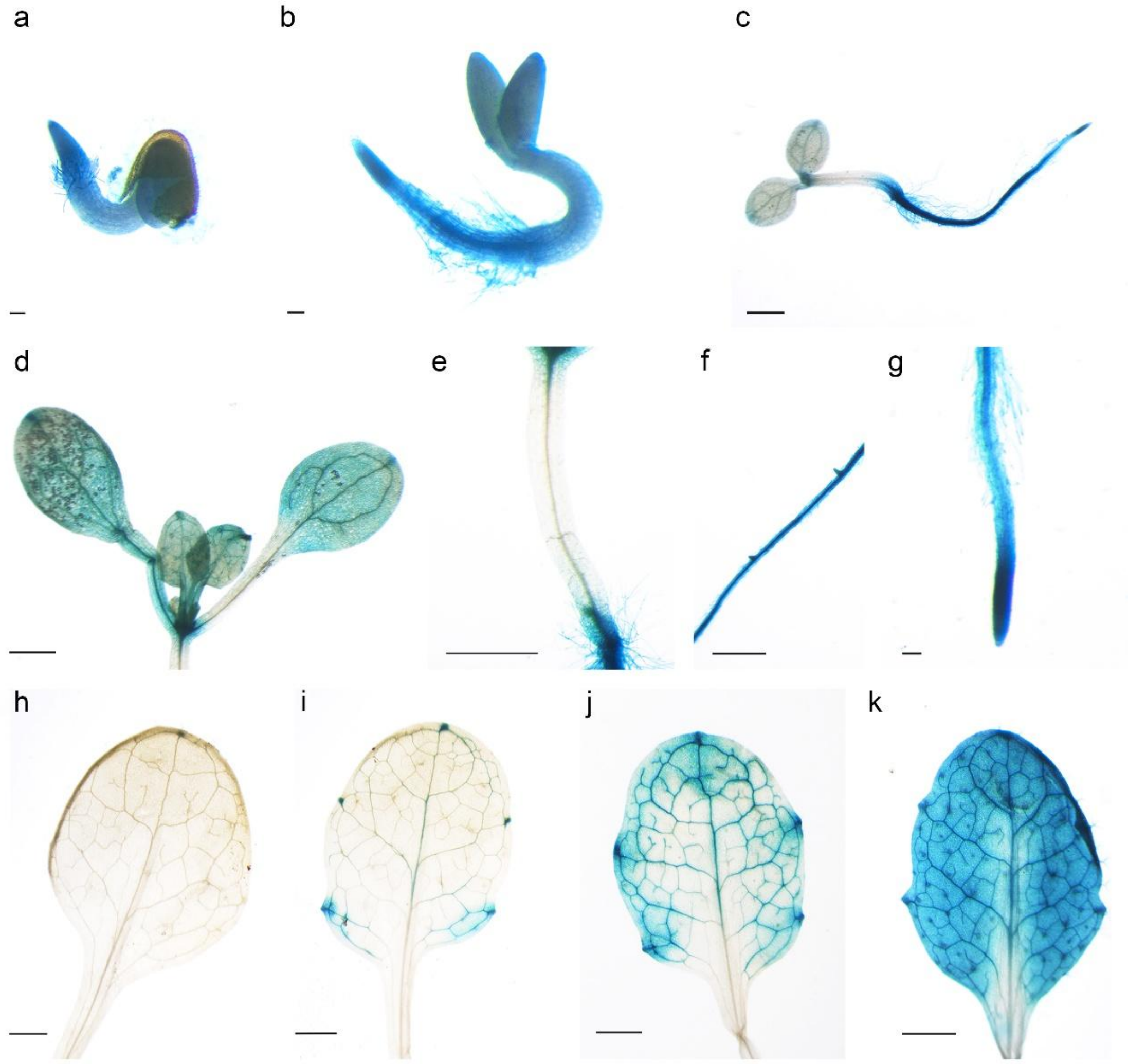

Figure S9. GUS histochemical staining in *AtPGD3::Pro-GUS* transgenic Arabidopsis seedlings.

Histochemical localization of GUS activity in **(a)** 1-day-old, **(b)** 2-day-old, **(c)** 5-day-old, **(d)** cotyledon, **(e)** transition zone, **(f)** root, and **(g)** root tip of 10-day-old, and **(h-k)** Leaves of 21-day-old *AtPGD3::Pro-GUS* transgenic Arabidopsis plants. Leaves ordered from oldest to youngest (left to right).

Scale bar in a, b, and g, 100μm. Scale bar in c-f, h-k, 1000 μm.

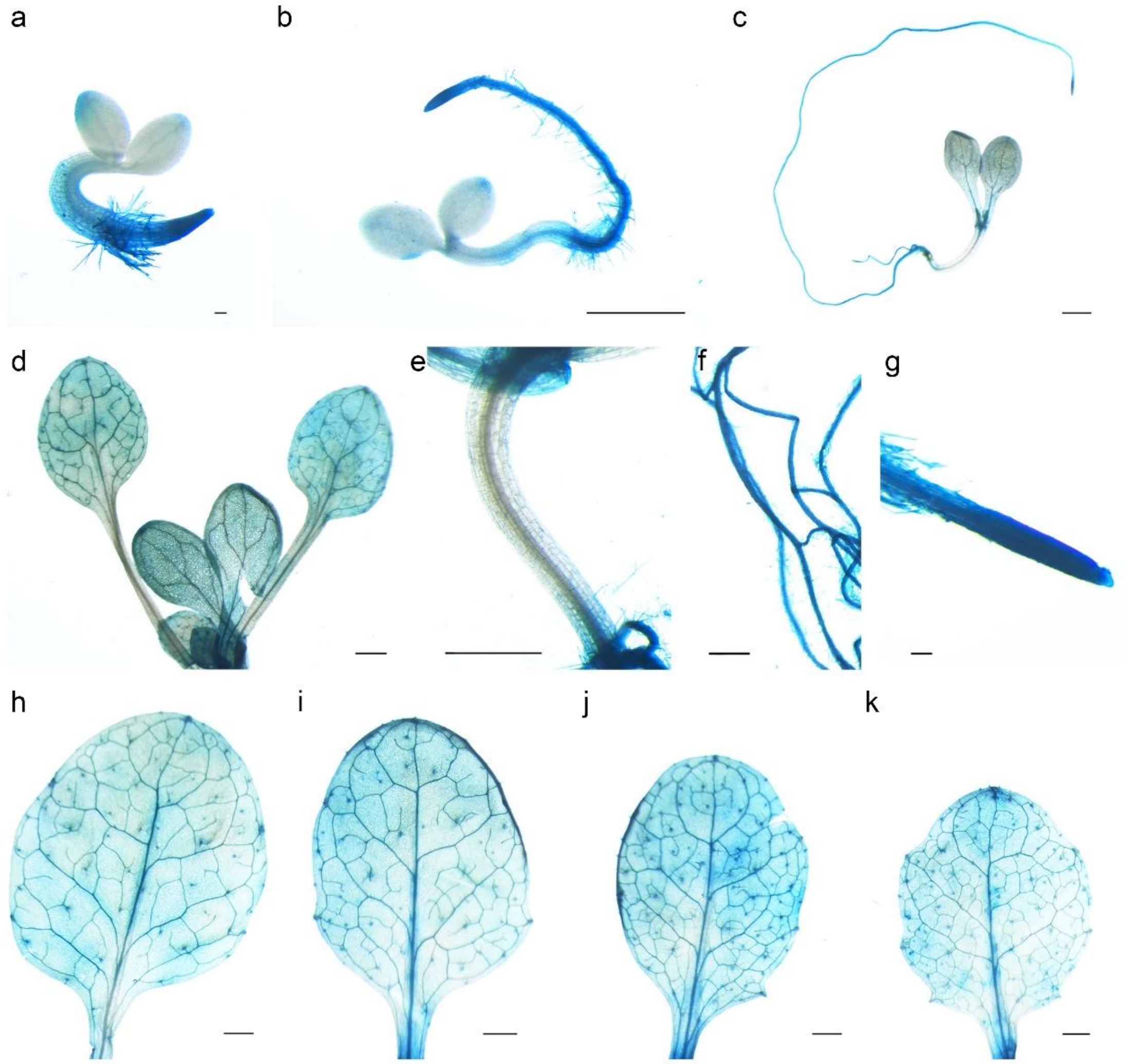

Figure S10. GUS histochemical staining in *AtcyNADP-ICDH::Pro-GUS* transgenic Arabidopsis seedlings.

Histochemical localization of GUS activity in **(a)** 1-day-old, **(b)** 2-day-old, **(c)** 5-day-old, **(d)** cotyledon, **(e)** transition zone, **(f)** root, and **(g)** root tip of 10-day-old, and **(h-k)** Leaves of 21-day-old *AtcyNADP-ICDH::Pro-GUS* transgenic Arabidopsis plants. Leaves ordered from oldest to youngest (left to right).

Scale bar in a, and g, 100μm. Scale bar in b-f, h-k, 1000 μm.

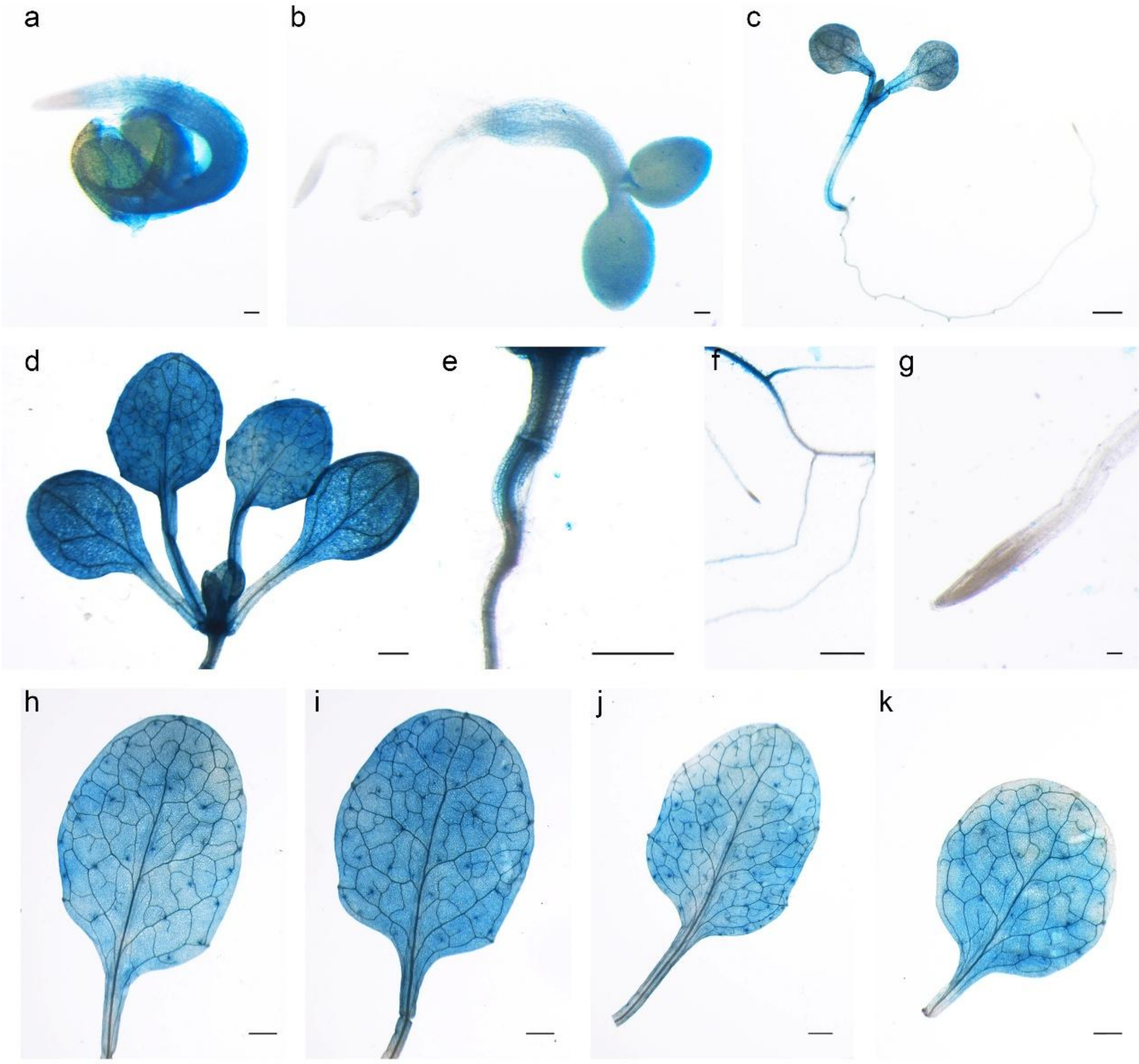

Figure S11. GUS histochemical staining in *Atnp-GAPDH::Pro-GUS* transgenic Arabidopsis seedlings.

Histochemical localization of GUS activity in **(a)** 1-day-old, **(b)** 2-day-old, **(c)** 5-day-old, **(d)** cotyledon, **(e)** transition zone, **(f)** root, and **(g)** root tip of 10-day-old, and **(h-k)** Leaves of 21-day-old *Atnp-GAPDH::Pro-GUS* transgenic Arabidopsis plants. Leaves ordered from oldest to youngest (left to right).

Scale bar in a, b, and g, 100μm. Scale bar in c-f, h-k, 1000 μm.

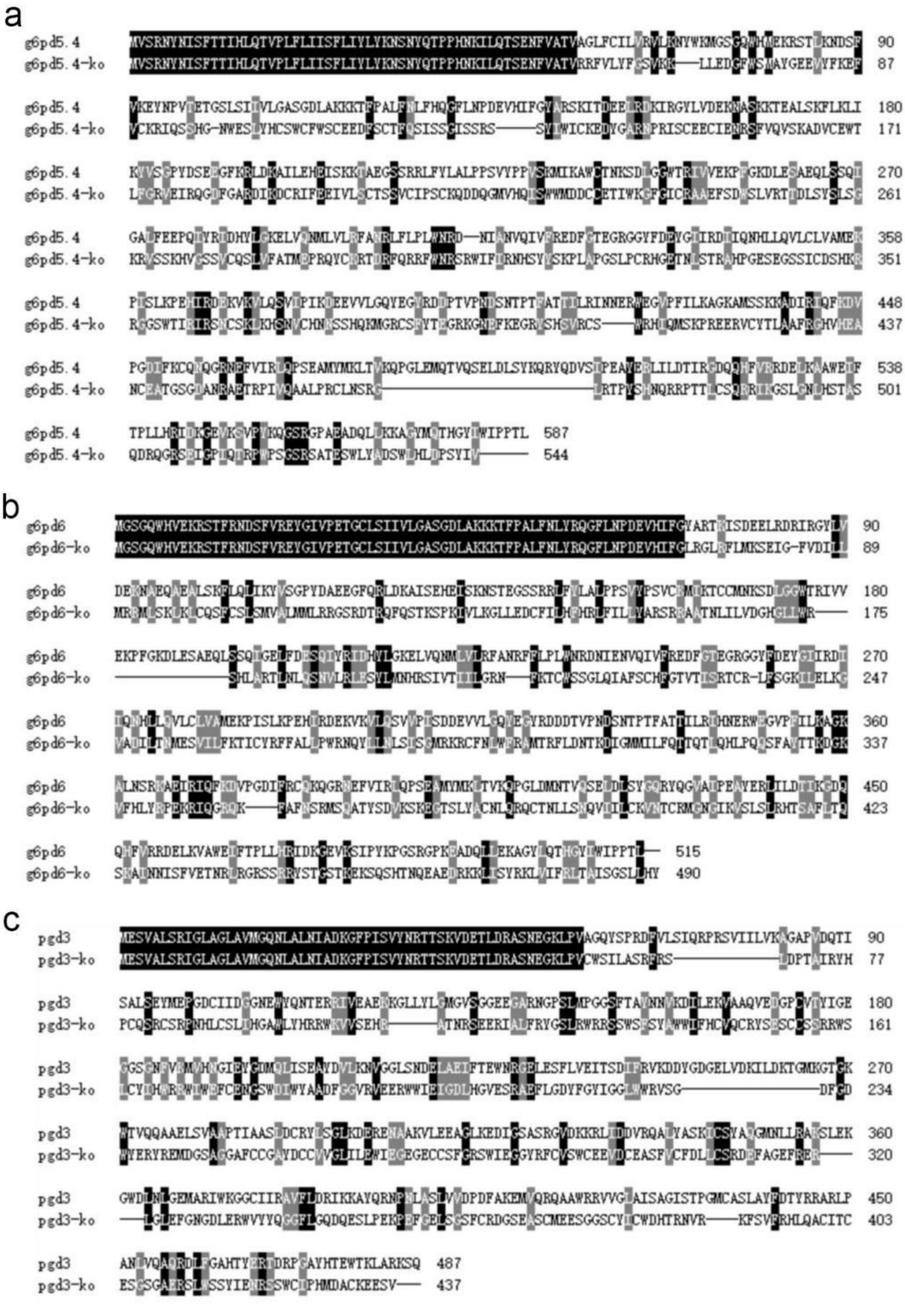

Figure S12. Affected target protein sequences of CRISPR/Cas9 lines.

(a) g6pd5.4 and g6pd5.4-ko denote the protein sequences derived from WT and *g6pd5.4* mutant, respectively. **(b)** g6pd6 and g6pd6-ko denote the protein sequences derived from WT and *g6pd6* mutant, respectively. **(c)** pgd3 and pgd3-ko denote the protein sequences derived from WT and *pgd3* mutant, respectively.

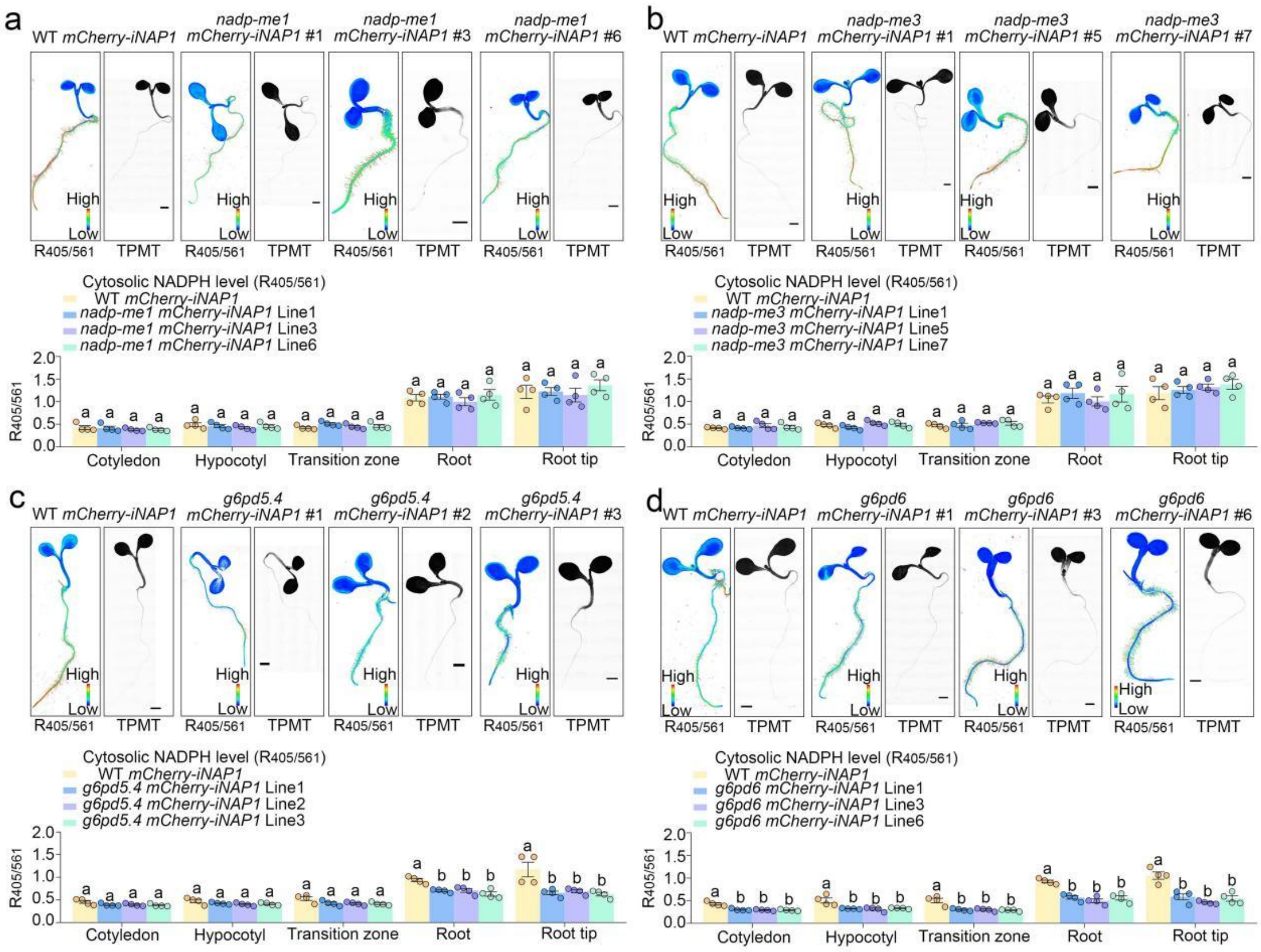

Figure S13. $R_{405/561}$ ratios in 5-day-old WT, *nadp-me1*, *nadp-me3*, *g6pd5.4* and *g6pd6* seedlings constitutively expressing mCherry-iNAP1 in the light.

Comparison of ratio images and bright-field images, and $R_{405/561}$ ratios of 5-day-old **(a)** *nadp-me1*, **(b)** *nadp-me3*, **(c)** *g6pd5.4* or **(d)** *g6pd6* seedlings expressing *mCherry-iNAP1* with WT *mCherry-iNAP1* plants. Three independent lines expressing mCherry-iNAP1 were characterized for each mutant genotype. In each line, $R_{405/561}$ ratios of 5 different tissues from 4 seedlings (n = 4) were subjected to data analysis, errors bar ± SEM, scale bar = 1000 µm. Fluorescence from sequential excitation wavelength at 405 nm and 561 nm, and the emission was collected at 520 ± 16 nm or 609 ± 25 nm, respectively. Ratio images are presented in pseudocolor, with red and blue representing high and low $R_{405/561}$ ratios (indicative of high and low NADPH levels), respectively. The significant differences in cytosolic NADPH levels were determined by One-way ANOVA with Tukey's HSD ($p < 0.05$) and indicated with different letters. TPMT, transmitted light detector; WT, wild-type.

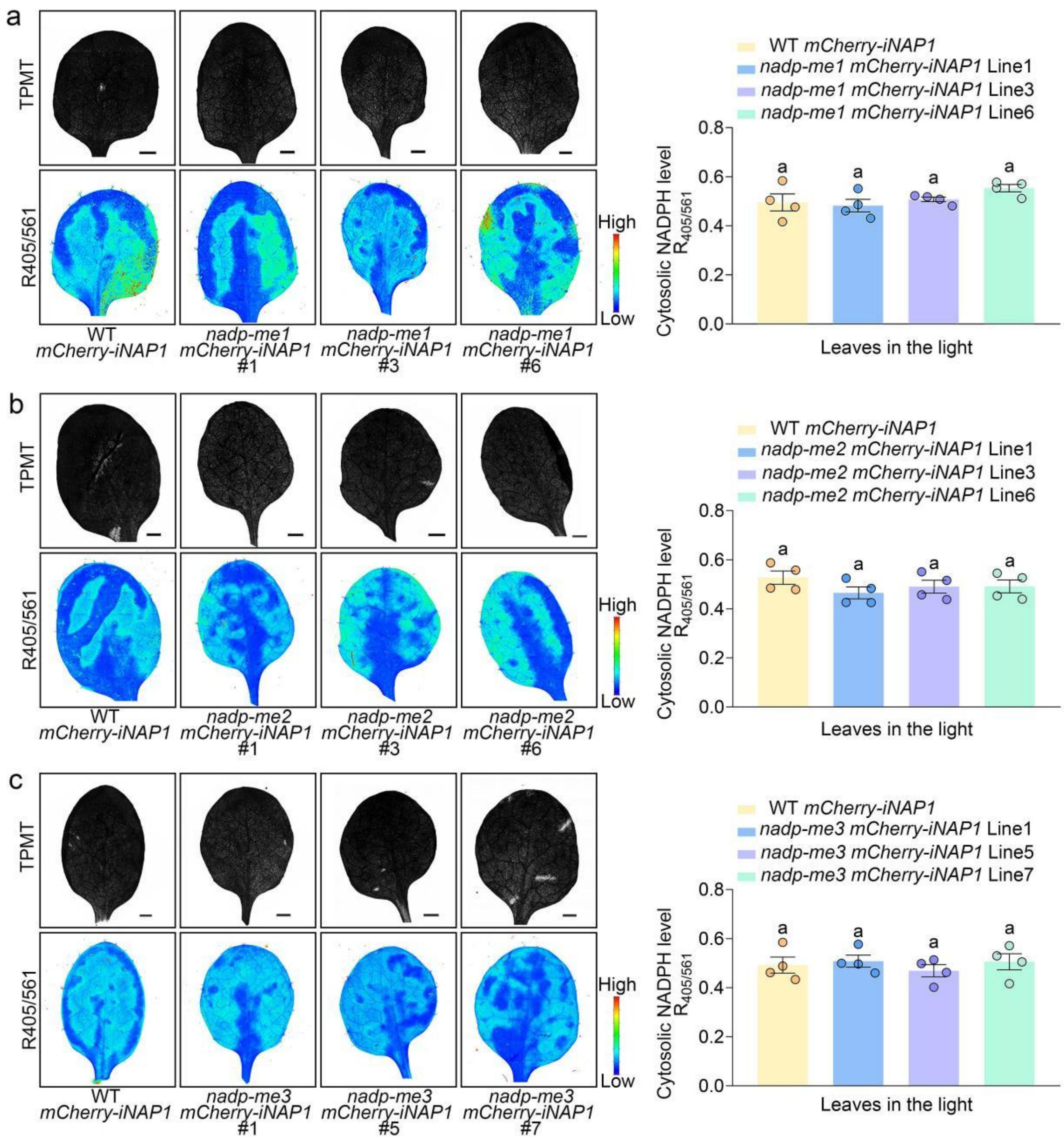

Figure S14. $R_{405/561}$ ratios in fully expanded leaves of 21-day-old WT, *nadp-me1*, *nadp-me2* and *nadp-me3* seedlings constitutively expressing *mCherry-iNAP1* in the light.

Comparison of ratio images and bright-field images, and $R_{405/561}$ ratios of fully expanded leaves in 21-day-old **(a)** *nadp-me1*, **(b)** *nadp-me2*, or **(c)** *nadp-me3* seedlings expressing *mCherry-iNAP1* with WT *mCherry-iNAP1* in the light. Three independent lines expressing mCherry-iNAP1 were characterized for each mutant genotype. For each column, *n* = 4 fully expanded leaves (L3 or L4) from four different seedings, errors bar ± SEM, scale bar = 1000 μm. Fluorescence from sequential excitation wavelength

at 405 nm and 561 nm, and the emission was collected at 520 ± 16 nm or 609 ± 25 nm, respectively. Ratio images are presented in pseudocolor, with red and blue representing high and low $R_{405/561}$ ratios (indicative of high and low NADPH levels), respectively. The significant differences in cytosolic NADPH levels were determined by One-way ANOVA with Tukey's HSD ($p < 0.05$) and indicated with different letters. TPMT, transmitted light detector; WT, wild-type.

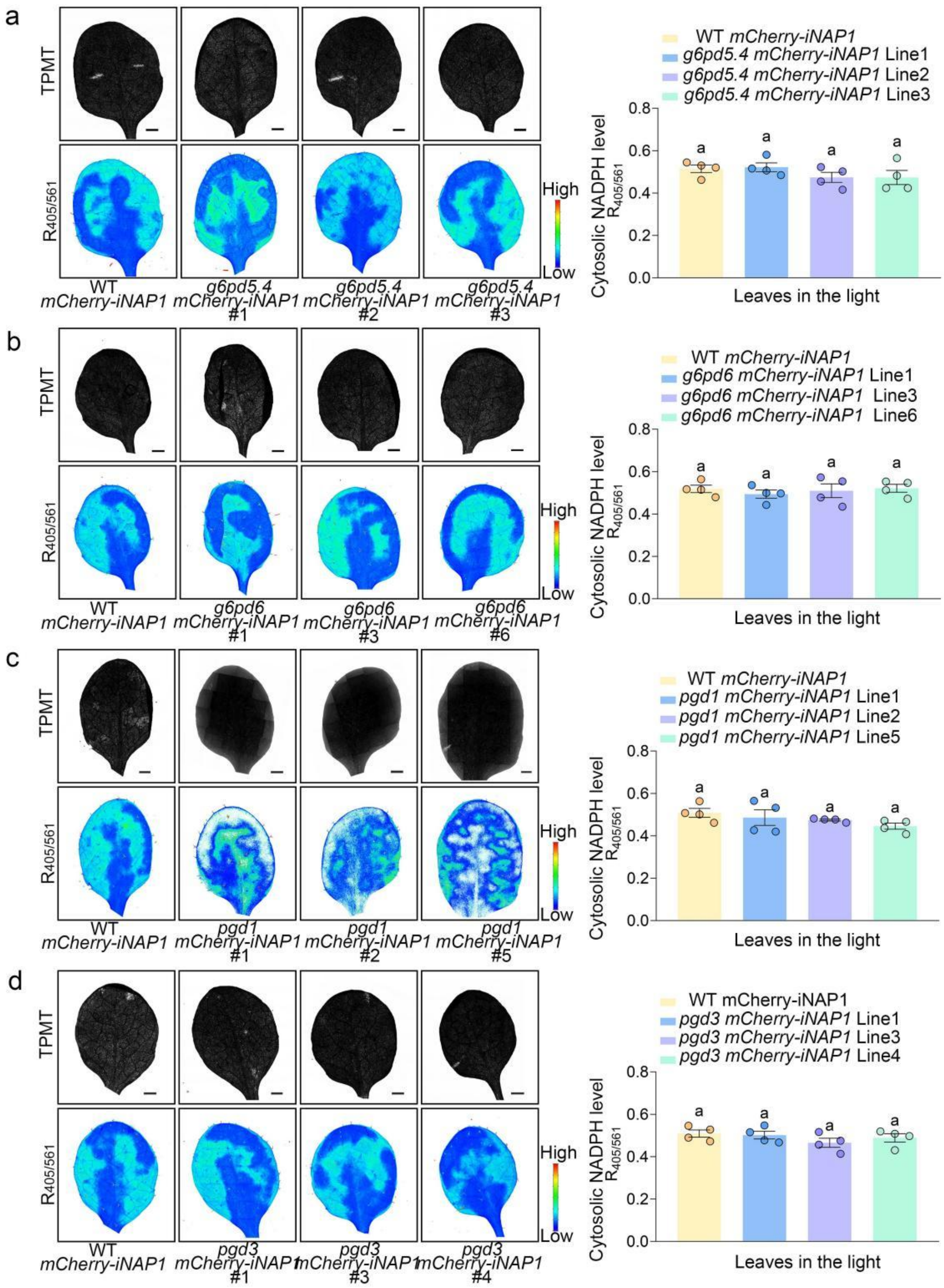

Figure S15. $R_{405/561}$ ratios in fully expanded leaves of 21-day-old WT, *g6pd5.4*, *g6pd6*, *pgd1* and *pgd3* seedlings constitutively expressing *mCherry-iNAP1* in the light.

Comparison of ratio images and bright-field images, and $R_{405/561}$ ratios of fully expanded leaves in 21-day-old **(a)** *g6pd5.4*, **(b)** *g6pd6*, **(c)** *pgd1*, and **(d)** *pgd3* seedlings expressing *mCherry-iNAP1* with WT *mCherry-iNAP1* in the light. Three independent lines expressing mCherry-iNAP1 were characterized for each mutant genotype. For each column, *n* = 4 fully expanded leaves (L3 or L4) from four different seedings, errors

bar ± SEM, scale bar = 1000 μm. Fluorescence from sequential excitation wavelength at 405 nm and 561 nm, and the emission was collected at 520 ± 16 nm or 609 ± 25 nm, respectively. Ratio images are presented in pseudocolor, with red and blue representing high and low $R_{405/561}$ ratios (indicative of high and low NADPH levels), respectively. The significant differences in cytosolic NADPH levels were determined by One-way ANOVA with Tukey's HSD ($p < 0.05$) and indicated with different letters. TPMT, transmitted light detector; WT, wild-type.

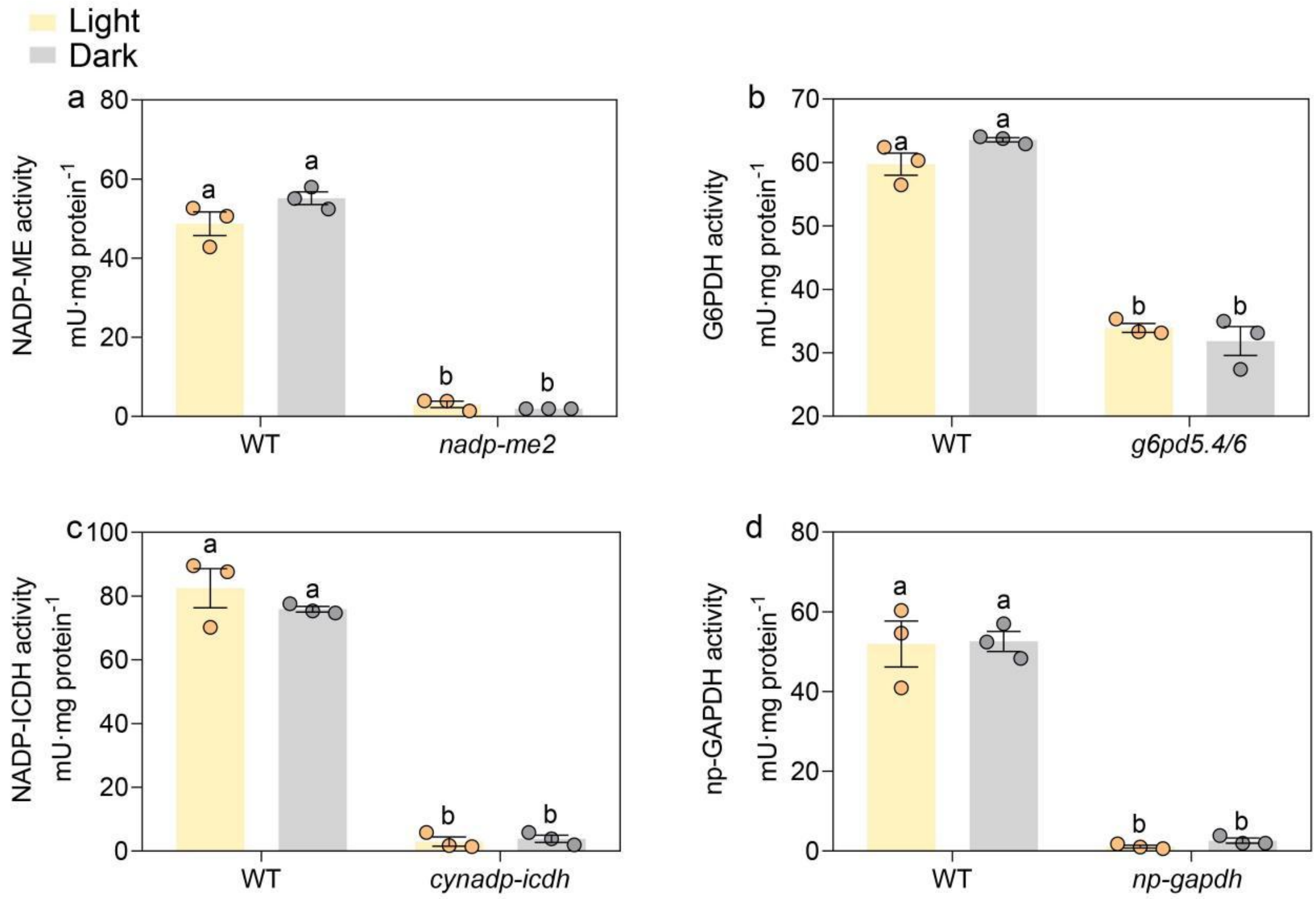

Figure S16. Enzyme activities of NADP-ME, G6PDH, NADP-ICDH and np-GAPDH in the cytosolic fractions of 21-day-old leaves of WT and mutants under light and dark conditions. (a) NADP-ME activities in the cytosolic fractions of WT and *nadp-me2* leaves under light and dark conditions, **(b)** G6PDH activities in the cytosolic fractions of WT and *g6pd5.4/6* leaves under light and dark conditions, **(c)** NADP-ICDH activities in the cytosolic fractions of WT and cy*nadp-icdh* leaves under light and dark conditions, **(d)** np-GAPDH activities in the cytosolic fractions of WT and *np-gapdh* leaves under light and dark conditions. One unit (U) of enzyme activity is defined as the generation of 1 μmol NADPH per minute under the specific assay conditions of each enzyme as described in Material and methods. For each column, n = 3 biological replicates, errors bar ± SEM. The significant differences in enzyme activities were determined by One-way ANOVA with Tukey's HSD ($p < 0.05$) and indicated with different letters.

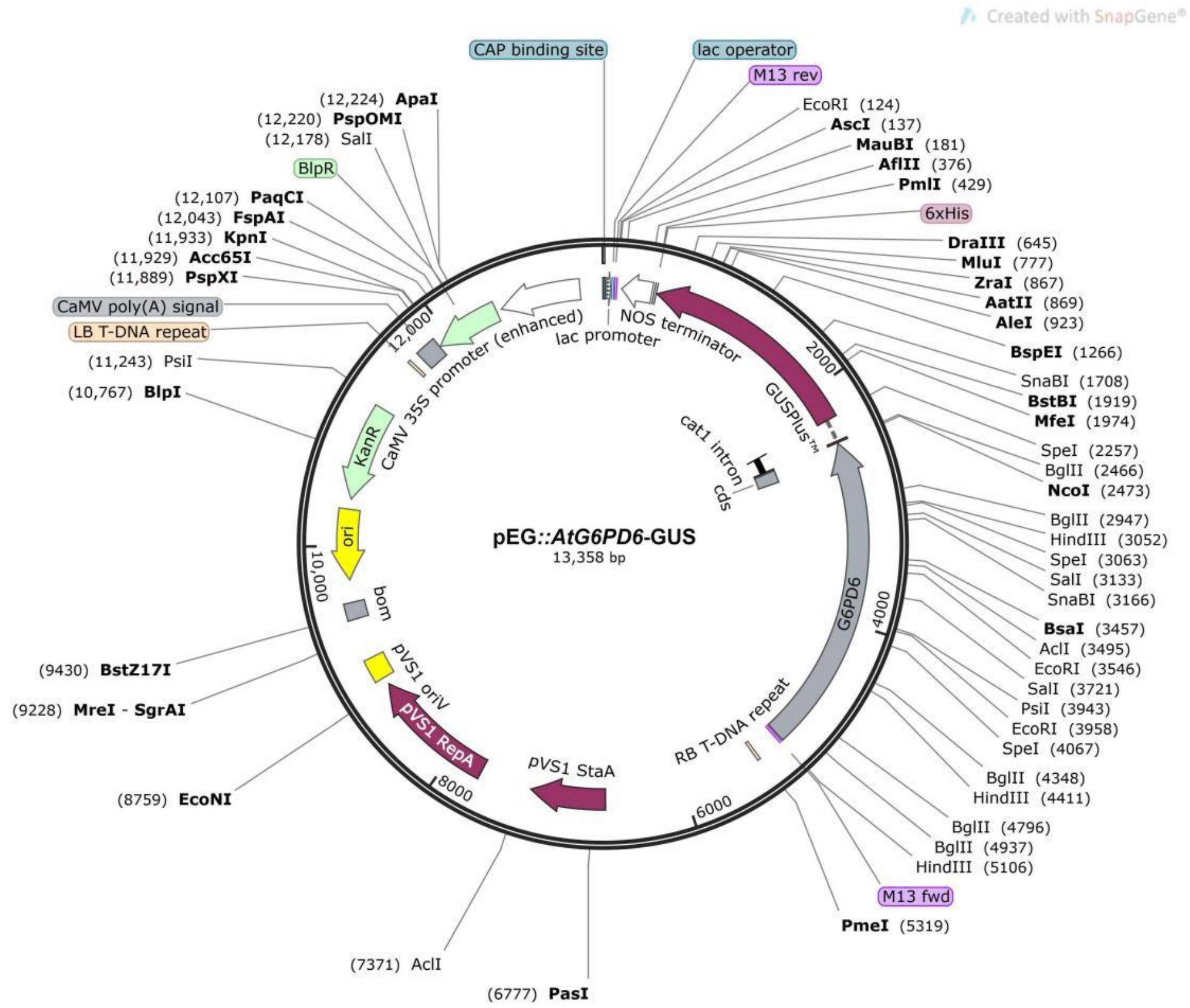

Figure S17. pEG*::AtG6PD6*-GUS vector map.

Figure obtained from EDGENE BIO company (Wuhan, China). BlpR, phosphinothricin acetyltransferase confers resistance to bialophos or phosphinothricin; Bom, basis of mobility region from pBR322; CaMV 35S promoter, CaMV, cauliflower mosaic virus 35S promoter; CAP binding site, *Escherichia. coli* catabolite activator protein; GUS, β-glucuronidase; G6PD6, promoter of glucose-6-phosphate dehydrogenase 6; KanR, minoglycoside phosphotransferase confers resistance to kanamycin; Lac operater, *lac* repressor encoded by *lacI*; LB, left border; NOS, nopaline synthase terminator and poly(A) signal; Ori, high-copy-number ColE1/pMB1/pBR322/pUC origin of replication; pVS1 oriv, origin of replication for the *Pseudomonas* plasmid pVS1; pVS1 RepA, replication protein from the *Pseudomonas* plasmid pVS1; pVS1 StaA, stability protein from the *Pseudomonas* plasmid pVS1; RB, right border. The *AtG6PD6* promoter region employed in this vector comprises 2,478 bp upstream of the first exon and extends to include the initial 141 bp of the first exon.

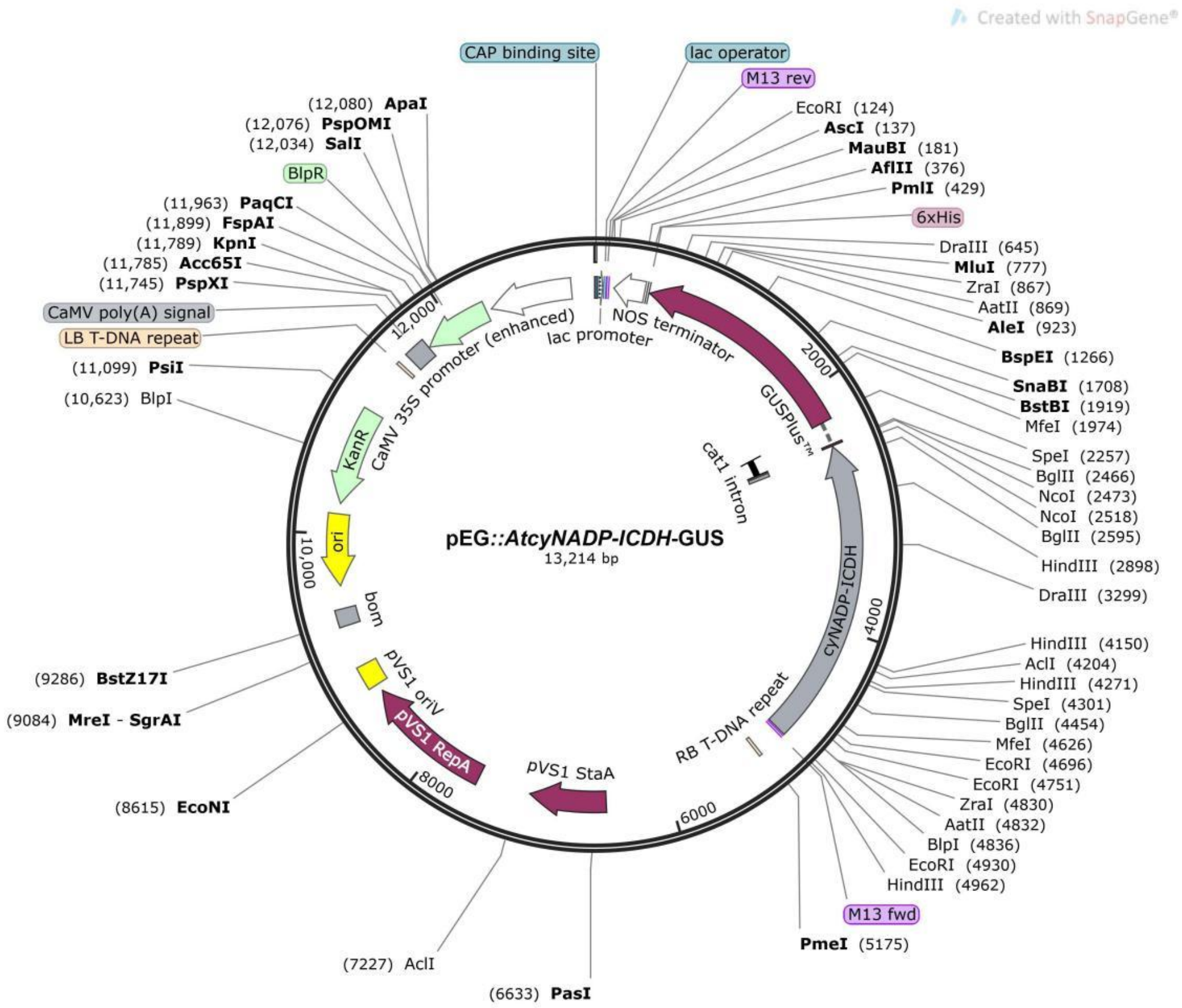

Figure S18. pEG*::AtcyNADP-ICDH*-GUS vector map.

Figure obtained from EDGENE BIO company (Wuhan, China). BlpR, phosphinothricin acetyltransferase confers resistance to bialophos or phosphinothricin; Bom, basis of mobility region from pBR322; CaMV 35S promoter, CaMV, cauliflower mosaic virus 35S promoter; CAP binding site, *Escherichia. coli* catabolite activator protein; GUS, β-glucuronidase; G6PD6, promoter of glucose-6-phosphate dehydrogenase 6; KanR, minoglycoside phosphotransferase confers resistance to kanamycin; Lac operater, *lac* repressor encoded by *lacI*; LB, left border; NOS, nopaline synthase terminator and poly(A) signal; Ori, high-copy-number ColE1/pMB1/pBR322/pUC origin of replication; pVS1 oriv, origin of replication for the *Pseudomonas* plasmid pVS1; pVS1 RepA, replication protein from the *Pseudomonas* plasmid pVS1; pVS1 StaA, stability protein from the *Pseudomonas* plasmid pVS1; RB, right border. The *AtcyNADP-ICDH* promoter region employed in this vector comprises the first 39 bp of the first exon and the 2436 bp upstream of the first exon.

Table S1. mRNA expression levels of genes encoding cytosolic NADPH generating enzymes and NADPH consuming enzymes in Col-0 Arabidopsis.

Pathway	Gene	Locus	Pollen	7-d root	17-d root	8-d seedling	21-d seedling	17-d Rosette leaves
Cytosolic NADPH generation pathway	*G6PD5*	AT3G27300	6.73	8.52	8.36	6.9	6.75	7.25
	G6PD6	AT5G40760	5.93	8.99	9.16	6.26	6.31	7.35
	PGD1	AT1G64190	6.86	10.93	10.59	8.61	8.46	9.03
	PGD2	AT3G02360	9.92	11.07	10.89	9.76	9.52	9.5
	PGD3	AT5G41670	7.41	10.79	10.29	8.51	8.05	9.06
	NADP-ME1	AT2G19900	5.78	4.8	4.7	5.27	5.19	5.2
	NADP-ME2	AT5G11670	6.65	11.42	11.75	11.36	11.08	10.39
	NADP-ME3	AT5G25880	9.3	4.46	4.6	4.59	4.51	4.79
	cyNADP-ICDH	AT1G65930	9.76	13.01	13.1	12.31	12.07	11.56
	np-GAPDH	AT2G24270	6.24	4.46	4.88	10.77	10.83	10.12
Cytosolic NADPH consuming pathway	*RboHA*	AT5G07390	4.84	5	4.51	3.8	3.82	3.8
	RboHB	AT1G09090	3.51	7.07	7.04	3.19	3.19	3.22
	RboHC	AT5G51060	5.2	7.42	7.16	4.49	4.35	4.45
	RboHD	AT5G47910	5.01	9.67	9.35	9.66	10.28	9.08
	RboHE	AT1G19230	3.98	4.91	4.74	3.77	3.85	3.65
	RboHF	AT1G64060	4.86	7.35	7.24	5.61	5.62	5.67
	RboHG	AT4G25090	5.16	6.59	5.55	4.31	4.33	4.31
	RboHH	AT5G60010	9.53	3.79	3.88	4.12	4.04	4.05
	RboHI	AT4G11230	3.42	4.49	4.3	3.22	3.24	3.26
	RboHJ	AT3G45810	6.84	3.64	3.62	3.79	3.66	3.8
	GR1	AT3G24170	8.44	9.09	9.19	9.04	8.55	8.67
	NTRA	AT2G17420	7.83	10.22	10.21	9.41	8.93	8.17
	NDB1	AT4G28220	5.21	6.52	6.66	6.32	5.99	7.37
	HPR2	AT1G79870	7.33	8.49	8.39	8.66	8.45	8.92
	ATR1	AT4G24520	7.52	9.36	9.51	9.11	9.02	8.5
	KCR1	AT1G67730	7.53	10.8	10.48	9.61	9.46	9.77
	ECR	AT3G55360	6.94	9.33	9.24	9.23	8.8	9.34

The microarray data were collected from *Qin Y, et al.*'s work (Qin et al., 2009). Data are presented as normalized log_2-transformed expression levels from 3-4 replicates (mean $log_2 = 6.15$).

Table S2. Catalytic properties of NADPH-generating enzymes.

Enzyme	Protein type	AGI code	K_{cat} (s^{-1})	K_m $NADP^+$(μM)	K_{cat}/K_m($NADP^+$)	K_m malate (μM)			Reference
AtNADP-ME1	recombinant protein	AT2G19900	38.70	205.10	0.19	2960.00			(Wheeler et al., 2005)
AtNADP-ME2	recombinant protein	AT5G11670	324.10	72.10	4.50	3330.00			
AtNADP-ME3	recombinant protein	AT5G25880	268.10	6.50	41.25	830.00			
			**K_{cat} (s^{-1})**	**K_m $NADP^+$(μM)**	**K_{cat}/K_m ($NADP^+$)**				
AtG6PD5	recombinant protein	AT3G27300	1.48	19.00	0.08				(Wakao and Benning, 2005)
AtG6PD6	recombinant protein	AT5G40760	98.33	6500.00	0.02				
			**K_{cat} (s^{-1})**	**K_m $NADP^+$(μM)**	**K_{cat}/K_m ($NADP^+$)**	**K_{cat}(s^{-1}) isocitrate**	**$K_m$isocitrate(μM)**	**K_{cat}/K_m (isocitrate)**	
AtcyNADP-ICDH	extracted from leaves	AT1G65930	87.75	4.40	19.94	63.90	42.00	1.52	(Leterrier et al., 2012)
	extracted from roots		232.35	61.60	3.77	202.20	239.00	0.85	
			**K_{cat} (s^{-1})**	**K_m $NADP^+$(μM)**	**K_{cat} / K_m ($NADP^+$)**	**K_{cat} (s^{-1}) GAP**	**K_m GAP (μM)**	**K_{cat} / K_m (GAP)**	
Atnp-GAPDH	recombinant protein	AT2G24270	5.50	25.60	0.21	5.40	125.20	0.04	(Piattoni et al., 2010)

The V_{max} (U mg^{-1}) values reported in original research papers were converted to K_{cat} (s^{-1}) using the Molecular Weight (MW). The MW of AtG6PD5-6 was 59 kDa (Wakao and Benning, 2005), the MW of AtcyNADP-ICDH was 90 kDa (Niazi et al., 2019), and the MW of Atnp-GAPDH was 60 kDa (Piattoni et al., 2010).

Table S3. Metabolites contents in leaves in the day and night (μM).

Metabolite	Organism	At the end of the day	At the middle of the night	Compartment	Reference
malate	Spinach	1000	500	cytosol	(Gerhardt et al., 1987)
	Spinach	29000	14000	vacuole	(Gerhardt et al., 1987)
	Arabidopsis	5800	2000	whole leaves	(Annunziata et al., 2018)
Sucrose	Spinach	5000	3500	cytosol	(Gerhardt et al., 1987)
	Spinach	22500	10000	vacuole	(Gerhardt et al., 1987)
G6P	Spinach	835	530	cytosol	(Gerhardt et al., 1987)
	Arabidopsis	120	90	whole leaves	(Annunziata et al., 2018)
citrate	Arabidopsis	2200	3200	whole leaves	(Annunziata et al., 2018)
isocitrate	Arabidopsis	32	36	whole leaves	(Annunziata et al., 2018)
DHAP	Spinach	80	10	cytosol	(Gerhardt et al., 1987)
3-PGA	Spinach	600	480	cytosol	(Gerhardt et al., 1987)

Chlorophyll content of spinach leaves was around 5 mg/g FW (Aslam et al., 2016). The metabolites concentrations in spinach leaves were converted from nmol·mg chl^{-1} to nmol·g FW^{-1}. Then convert nmol·g FW^{-1} to μM assuming 1 g fresh weight (FW) equals approximately 1 mL (Veljanovski et al., 2006). G6P, glucose 6-phosphate; DHAP, dihydroxyacetone phosphate; 3-PGA, 3-phosphoglycerate.

Table S4. mRNA expression levels of genes encoding cytosolic NADPH generating enzymes and NADPH consuming enzymes in Col-0 Arabidopsis in the 13th and 21st hours of the day (16 h light/8 h dark cycle).

	Gene	AGI code	13h	21h	Fold change	Log2|Fold
Cytosolic NADPH generation pathway	***G6PD5***	AT3G2730	**94.23**	**48.55**	**0.52**	**-0.96**
	G6PD6	AT5G4076	28.87	27.16	0.94	-0.09
	PGD1	AT1G6419	47.22	44.13	0.93	-0.10
	PGD2	AT3G0236	113.11	125.93	1.11	0.15
	PGD3	AT5G4167	73.20	79.87	1.09	0.13
	NADP-ME1	AT2G1990	0	0.024	-	-
	NADP-ME2	AT5G1167	**505.23**	**217.91**	**0.43**	**-1.21**
	cyNADP-	AT1G6593	**461.89**	**1356.6**	**2.94**	**1.55**
	np-GAPDH	AT2G2427	**171.28**	**307.42**	**1.79**	**0.84**
Cytosolic NADPH consumption pathway	*RboHC*	AT5G5106	1.16	0.95	0.82	-0.29
	RboHD	AT5G4791	99.79	95.83	0.96	-0.06
	RboHE	AT1G1923	0.52	0.36	0.71	-0.50
	RboHF	AT1G6406	28.32	24.54	0.87	-0.21
	GR1	AT3G2417	**105.14**	**190.73**	**1.81**	**0.86**
	NTRA	AT2G1742	78.86	99.53	1.26	0.34
	NDB1	AT4G2822	27.26	28.18	1.03	0.05
	HPR2	AT1G7987	91.73	73.84	0.80	-0.31
	ATR1	AT4G2452	**77.39**	**165.58**	**2.14**	**1.10**
	KCR1	AT1G6773	49.54	49.72	1.00	0.01
	ECR	AT3G5536	64.03	58.28	0.91	-0.14

The relative gene expression data were obtained and analyzed from *Jung S, et al* (Jung et al., 2024). RNA-seq data from *Jung S, et al.* were generated using total RNA isolated from the third and fourth leaves of 21-day-old Col-0 plants, grown in 16-h light/8-h dark cycle, at zeitgeber time 13 and 21 (three biological replicates each). The relative expression of genes was assessed and is presented as a percentage of the average RNA-seq reads. Counts per million normalized (CPM) normalized by trimmed mean of m-values (TMM). In the transcriptome data, *NADP-ME1*, *RboHA-B*, *RboHG-J* was not detected. Significant

differences of fold change values were determined by independent samples t-test ($P < 0.05$). Genes highlighted in bold (fold change > 1.5 or < 0.66, p-value < 0.5).

Table S5. Primer sequences used in this study.

Primer	Sequence (5'- 3')
**Primer sequences for promoter-GUS fusion construct**	
GUS_NADP-ME1 Pro_FW	5'-GACCATGATTACGCCAAGCTTCCTGACCTGTGAACCGGAATT-3'
GUS_NADP-ME1 Pro_RW	5'-GACTGACCACCCGGGGATCCGTAGCGAGGATCTCTCAGCA-3'
GUS_NADP-ME2 Pro_FW	5'-GACCATGATTACGCCAAGCTTGAGCTAAGCTGAACTGGATCT-3'
GUS_NADP-ME2 Pro_RW	5'-GACTGACCACCCGGGGATCCGGATCTGTTATCGGCGACATC-3'
GUS_NADP-ME3 Pro_FW	5'-GACCATGATTACGCCAAGCTTGATAACCGCCGTAAGCAGACG-3'
GUS_NADP-ME3 Pro_RW	5'-GACTGACCACCCGGGGATCCGTAACGTGGATCACGCATCAA-3'
GUS_G6PD5 Pro_FW	5'-GACCATGATTACGCCAAGCTTATCGGAATGTATTGCTTGCTC-3'
GUS_G6PD5 Pro_RW	5'-GACTGACCACCCGGGGATCCGTTACGATTGATTGATTGGT-3'
GUS_PGD1 Pro_FW	5'-GACCATGATTACGCCAAGCTTAACGGGAAGACGATGCCCAGA-3'
GUS_PGD1 Pro_RW	5'-GACTGACCACCCGGGGATCCGAGTCCGGCGAGACCGATTCG-3'
GUS_PGD2 Pro_FW	5'-GACCATGATTACGCCAAGCTTAGAGGTAACCTCGGATCGATG-3'
GUS_PGD2 Pro_RW	5'-GACTGACCACCCGGGGATCCTCCAGCTAGACCTATTCTTGT-3'
GUS_PGD3 Pro_FW	5'-GACCATGATTACGCCAAGCTTAGAGAGCTTAGGATGATGCTC-3'
GUS_PGD3 Pro_RW	5'-GACTGACCACCCGGGGATCCTTGTCCCATGACTGCGAGAC-3'
GUS_np-GADPH Pro_FW	5'-GACCATGATTACGCCAAGCTTGGCCTTGAACCAAGATAACTA-3'
GUS_np-GADPH Pro_RW	5'-GACTGACCACCCGGGGATCCTTCTCCATCAAGAATCTCAGC-3'
**Primer sequences for genotyping T-DNA insertion lines**	
SALK-036898C_LP (nadp-me1)	5'-CACTTGCAGAGCATACCTTCC-3'
SALK-036898C_RP (nadp-me1)	5'-TGCTTACCAGCAGCTAGAAGC-3'
SALK-020607_LP (nadp-me2)	5'-CCTTCCAAGACAAGTTGATCG-3'
SALK-020607_RP (nadp-me2)	5'-TGCTGAACAGGCTTACACATG-3'
SALK-139336_LP (nadp-me3)	5'-CGGCAAAGAAATGAGTAATGG-3'
SALK-139336_RP (nadp-me3)	5'-GACCCTGTTCAGTATGATGGC-3'
SALK_056247_LP (cynadp-icdh)	5'-GCGTTTGAGAAGATCAAGGTG-3'
SALK_056247_RP (cynadp-icdh)	5'-ATCAGTGGCACGGTACTGATC-3'
LBb1.3 for SALK lines	5'-ATTTTGCCGATTTCGGAAC-3'
GABI_762C06_LP (pgd1)	5'-TATGGGTGGTTCTTGTCAAGG-3'
GABI_762C06_RP (pgd1)	5'-ACCGTTACGAGCTCCTTCTTC-3'
LB for GABI lines	5'-ATATTGACCATCATACTCATTGC-3'
SAIL_1152_B02_LP (np-gapdh)	5'-CTCTTGACAAAACGTTGCTCC-3'
SAIL_1152_B02_RP (np-gapdh)	5'-TTGGTGAAAGGTTGTGAGGTC-3'
LB1 for SAIL lines	5'-GCCTTTTCAGAAATGGATAAATAGCCTTGCTTCC-3'
**Primer sequences for semi-PCR**	
np-GAPDH-semi_FW	5'-AAAGCTCCCATGGCTGAGTC-3'
np-GAPDH-semi_RW	5'-AGTACGGTCATTACCGGGGA-3'
act8-semi_FW	5'-TTGGAATGGAAGCTGCAGGG-3'
act8-semi_RW	5'-AATGCCTGGACCTGCTTCAT-3'
**Primer sequences for single guide RNA**	
G6PD5_sgRNA_FW	5'-TAGGAATTTTGTCGCCACCGTCGC-3'
G6PD5_sgRNA_RW	5'-AAACGCGACGGTGGCGACAAAATT-3'
G6PD6_sgRNA_FW	5'-TAGGTTCATATTTTCGGGTATGCG-3'

G6PD6_sgRNA_RW	5'-AAACCGCATACCCGAAAATATGAA-3'
PGD3_sgRNA_FW	5'-ATTGCGAGTATTGACCAGCGAC-3'
PGD3_sgRNA_FW	5'-AAACGTCGCTGGTCAATACTCG-3'
**Primer sequences for PCR products sequencing**	
G6PD5_Cas9_Seq_FW	5'-CCTTGTTGGGCAGGTGAA-3'
G6PD5_Cas9_Seq_RW	5'-AACCAGCTTTAGAAACTTGGAC-3'
G6PD6_Cas9_Seq_FW	5'-GAAACCGAGGCTGTGAAT-3'
G6PD6_Cas9_Seq_RW	5'-ACAACAATCCGTGTCCAT-3'
PGD3_Cas9_Seq_FW	5'-GGCGTCGTAAATAAAGAGC-3'
PGD3_Cas9_Seq_RW	5'-TTTAGCAGCATTCTCCCTC-3'

References

Annunziata, M. G., Apelt, F., Carillo, P., et al. 2018. "Response of Arabidopsis primary metabolism and circadian clock to low night temperature in a natural light environment." *J Exp Bot* 69(20), 4881-4895.

Aslam, M., Sultana, B., Anwar, F., et al. 2016. "Foliar spray of selected plant growth regulators affected the biochemical and antioxidant attributes of spinach in a field experiment." *Turkish Journal of Agriculture and Forestry* 40, 136-145.

Gerhardt, R., Stitt, M., and Heldt, H. W. 1987. "Subcellular Metabolite Levels in Spinach Leaves: Regulation of Sucrose Synthesis during Diurnal Alterations in Photosynthetic Partitioning." *Plant Physiol* 83(2), 399-407.

Jung, S., Kim, H., Lee, J., et al. 2024. "The genetically programmed rhythmic alteration of diurnal gene expression in the aged Arabidopsis leaves." *Front Plant Sci* 15, 1481682.

Leterrier, M., Barroso, J. B., Palma, J. M., et al. 2012. "Cytosolic NADP-isocitrate dehydrogenase in Arabidopsis leaves and roots." *Biologia plantarum* 56(4), 705-710.

Niazi, A. K., Bariat, L., Riondet, C., et al. 2019. "Cytosolic Isocitrate Dehydrogenase from Arabidopsis thaliana Is Regulated by Glutathionylation." *Antioxidants (Basel)* 8(1), 16.

Piattoni, C. V., Rius, S. P., Gomez-Casati, D. F., et al. 2010. "Heterologous expression of non-phosphorylating glyceraldehyde-3-phosphate dehydrogenase from Triticum aestivum and *Arabidopsis thaliana*." *Biochimie* 92(7), 909-913.

Qin, Y., Leydon, A. R., Manziello, A., et al. 2009. "Penetration of the stigma and style elicits a novel transcriptome in pollen tubes, pointing to genes critical for growth in a pistil." *PLoS Genet* 5(8), e1000621.

Veljanovski, V., Vanderbeld, B., Knowles, V. L., et al. 2006. "Biochemical and molecular characterization of AtPAP26, a vacuolar purple acid phosphatase up-regulated in phosphate-deprived Arabidopsis suspension cells and seedlings." *Plant Physiol* 142(3), 1282-1293.

Wakao, S., and Benning, C. 2005. "Genome-wide analysis of glucose-6-phosphate dehydrogenases in Arabidopsis." *Plant J* 41(2), 243-256.

Wheeler, M. C., Tronconi, M. A., Drincovich, M. F., et al. 2005. "A comprehensive analysis of the NADP-malic enzyme gene family of Arabidopsis." *Plant Physiol* 139(1), 39-51.
